# Characterization of a new fusicoccane-type diterpene synthase and an associated P450 enzyme

**DOI:** 10.3762/bjoc.18.144

**Published:** 2022-10-05

**Authors:** Jia-Hua Huang, Jian-Ming Lv, Liang-Yan Xiao, Qian Xu, Fu-Long Lin, Gao-Qian Wang, Guo-Dong Chen, Sheng-Ying Qin, Dan Hu, Hao Gao

**Affiliations:** 1 Institute of Traditional Chinese Medicine & Natural Products, College of Pharmacy / Guangdong Province Key Laboratory of Pharmacodynamic Constituents of TCM and New Drugs Research / International Cooperative Laboratory of Traditional Chinese Medicine Modernization and Innovative Drug Development of Ministry of Education (MOE) of China, Jinan University, Guangzhou 510632, China; 2 Clinical Experimental Center, First Affiliated Hospital of Jinan University, Guangzhou 510630, China; 3 Shenzhen Institute of Synthetic Biology, Shenzhen Institute of Advanced Technology, Chinese Academy of Sciences, Shenzhen 518055, China

**Keywords:** cytochrome P450 enzyme, diterpene synthase, gene cluster, genome mining, site-directed mutagenesis

## Abstract

Fusicoccane-type terpenoids are a subgroup of diterpenoids featured with a unique 5-8-5 ring system. They are widely distributed in nature and possess a variety of biological activities. Up to date, only five fusicoccane-type diterpene synthases have been identified. Here, we identify a two-gene biosynthetic gene cluster containing a new fusicoccane-type diterpene synthase gene *tadA* and an associated cytochrome P450 gene *tadB* from *Talaromyces wortmannii* ATCC 26942. Heterologous expression reveals that TadA catalyzes the formation of a new fusicoccane-type diterpene talaro-7,13-diene. D_2_O isotope labeling combined with site-directed mutagenesis indicates that TadA might employ a different C2,6 cyclization strategy from the known fusicoccane-type diterpene synthases, in which a neutral intermediate is firstly formed and then protonated by an environmental proton. In addition, we demonstrate that the associated cytochrome P450 enzyme TadB is able to catalyze multiple oxidation of talaro-7,13-diene to yield talaro-6,13-dien-5,8-dione.

## Introduction

Terpenoids are a large class of natural products that attract extensive attention, due to not only their potential applications in pharmaceuticals, agrochemicals, etc. but also due to their abundant structural architectures [1]. Fusicoccane (FC)-type terpenoids are a subgroup of diterpenoids possessing a unique 5-8-5 tricyclic skeleton, which can be produced by plants, fungi and bacteria [2]. This type of diterpenoids, represented by fusicoccin A and cotylenin A, can serve as efficient modulators of 14-3-3 protein–protein interactions (PPIs) [3–4]. 14-3-3 PPIs, which refer to the binding interactions of 14-3-3 proteins with hundreds of “client” proteins, are associated with many diseases, such as cancer and neurodegenerative diseases [3–4]. As a result, lots of attempts, including traditional isolation from nature [5–8] and chemical synthesis [9], have been made to expand the structural diversity of FC-type diterpenoids for drug development.

**Figure 1:**
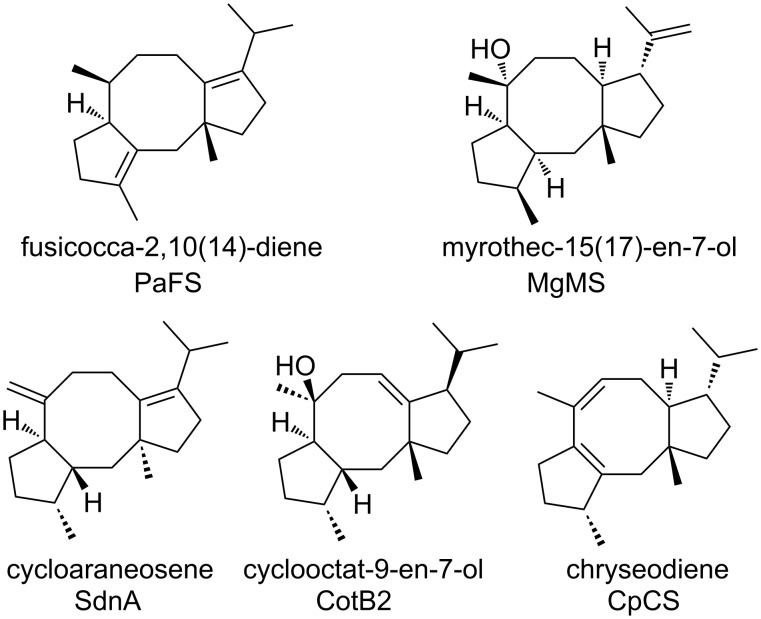
The five distinct FC-type DTSs and the corresponding products.

Along with the development of low-cost sequencing technologies and tractable heterologous expression systems, genome mining has become a promising strategy for targeted discovery of natural products [10–12], which can also provide enzymatic tools toward combinatorial biosynthesis [13–14]. As terpene synthases play a fundamental role in constructing molecular skeletons, great efforts have been devoted to mining novel synthases in pursuit of new terpenoids [15–17]. It is generally accepted that the FC-type diterpene skeleton is formed from geranylgeranyl diphosphate (GGPP) via a concerted C1,11–C10,14-bicyclization, followed by a C2,6-cyclization [18–21]. Theoretically, according to the configuration of stereogenic centers at C2, C6, C10, and C14 introduced during the two cyclization steps, FC-type diterpene synthases (DTSs) could be divided into 16 subtypes [20]. Furthermore, considering the modes of potential carbocation rearrangement and final carbocation quenching, there might be more FC-type DTSs in nature. To date, only five distinct FC-type DTSs, including fungi-derived PaFS/SdnA/MgMS and bacteria-derived CotB2/CpCS, have been reported (Figure 1) [20–24], implying that there still exists a large enzymatic space remaining to be explored.

Herein, we characterize a two-gene cluster from *Talaromyces wortmannii* ATCC 26942, in which TadA is identified to be a new FC-type DTS responsible for the formation of talaro-7,13-diene, and the associated P450 enzyme TadB is characterized to be a multifunctional enzyme, converting talaro-7,13-diene to highly oxygenated talaro-6,13-dien-5,8-dione.

## Results and Discussion

### Identification of a new fusicoccane-type DTS and an associated P450 enzyme

**Figure 2:**
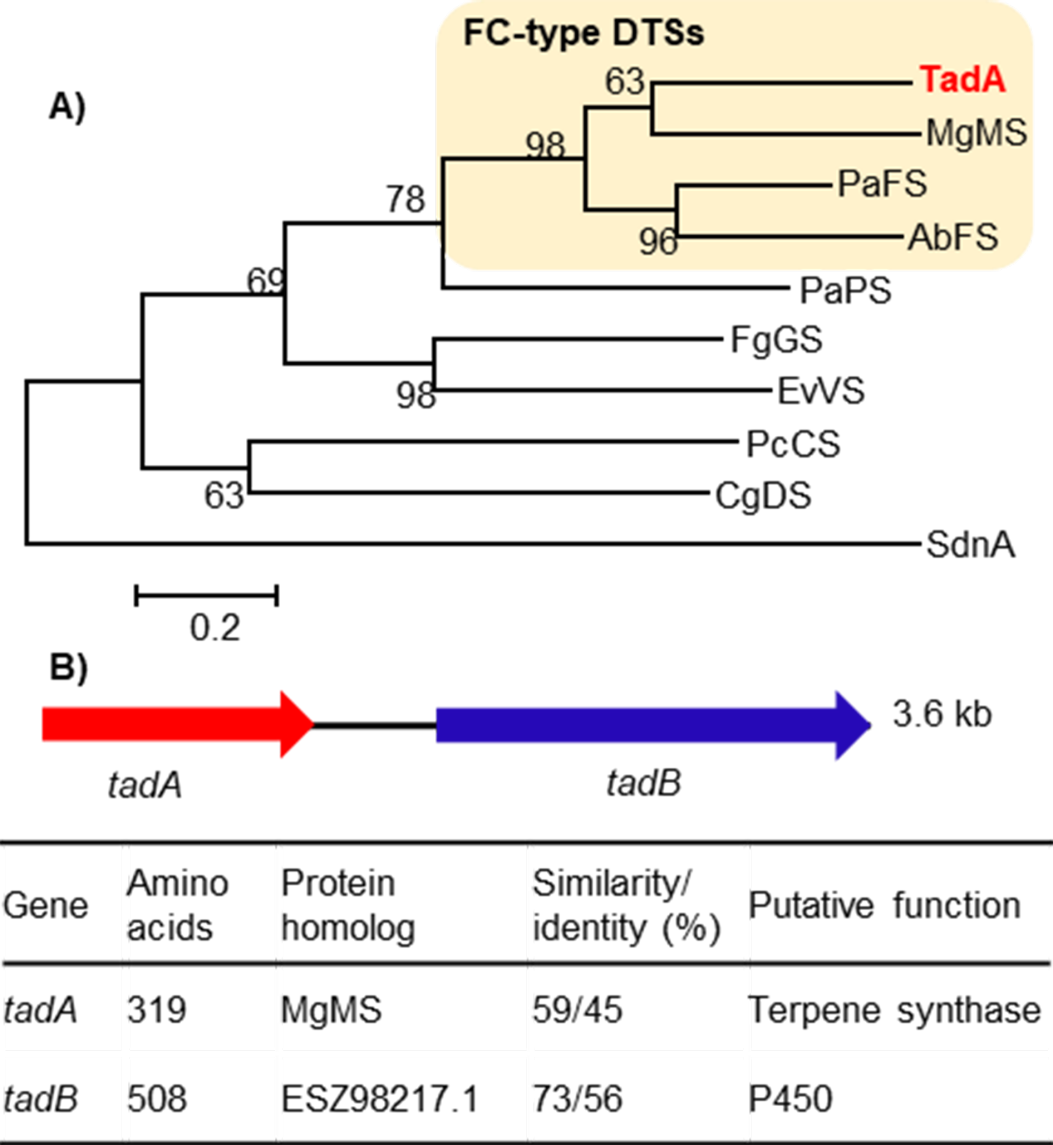
Bioinformatics analysis of the *tad* cluster. A) Phylogenetic tree of TadA and representative fungal DTSs. B) Putative functions of the *tad* cluster.

We used MgMS, a previously identified FC-type DTS in our group [20], as a query to perform local BLAST search against our in-house fungal genome database, and then found a candidate enzyme TadA from *T. wortmannii* ATCC 26942. Further phylogenetic analysis of TadA and representative fungal DTSs showed that TadA falls within the clade of FC-type DTSs (Figure 2A). In light of showing low amino acid sequence identity (<50%) to reported fungal FC-type DTSs [20,22,24–25], TadA was annotated as a putative new FC-type DTS. We then scanned the flanking region of *tadA*, and found a cytochrome P450 gene *tadB*. Accordingly, the two-gene cluster was termed *tad* cluster (Figure 2B), and the sequence data was deposited in GenBank under the accession number ON624151.

### Functional analysis of the FC-type DTS TadA

**Figure 3:**
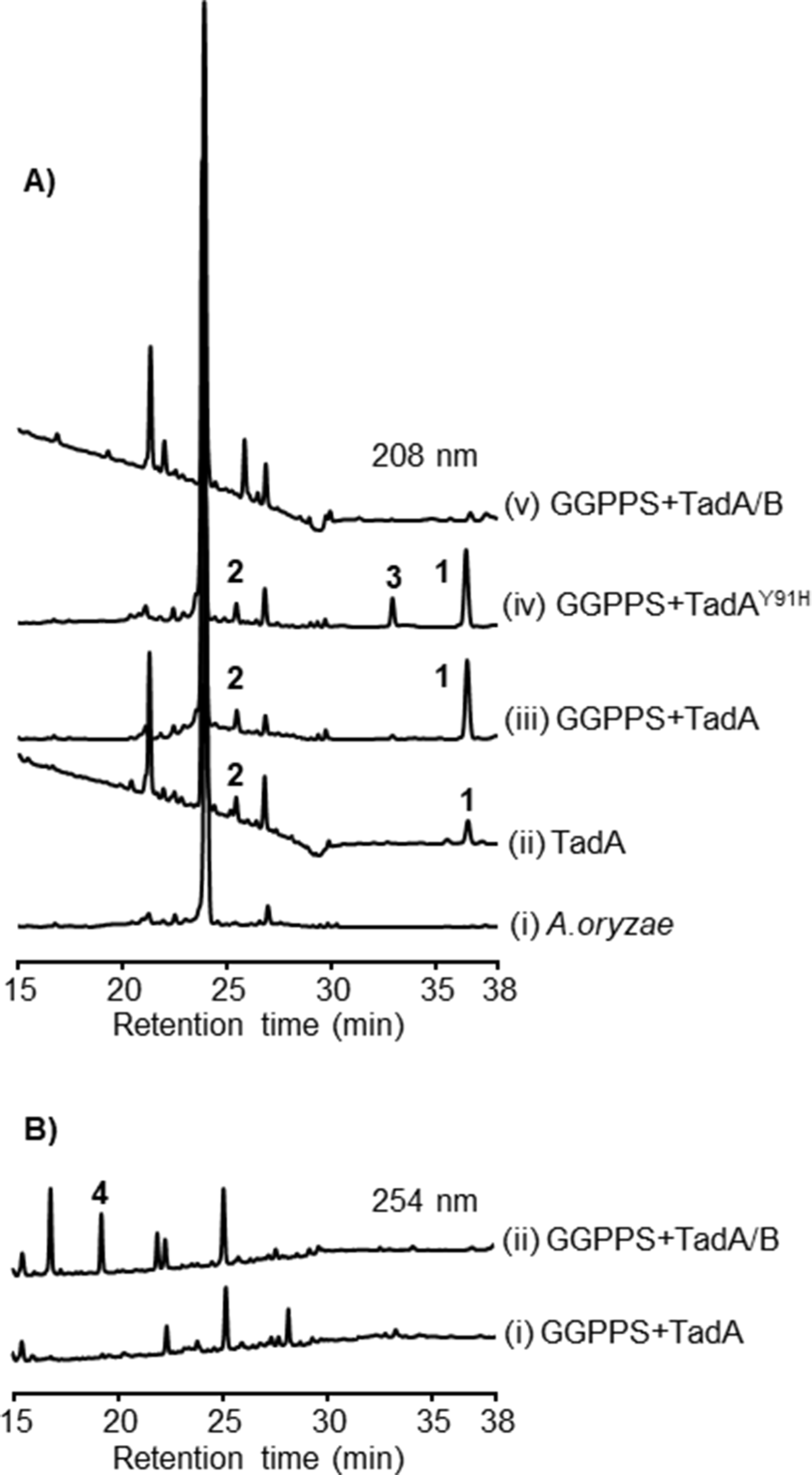
HPLC–MS analysis of mycelial extracts from *A. oryzae* NSAR1 transformants. A) The HPLC profiles monitored at 208 nm. B) The HPLC profiles monitored at 254 nm.

In order to analyze the function of TadA, we introduced *tadA* into the quadruple auxotrophic host *Aspergillus oryzae* NSAR1 (*niaD^−^*, *sC^−^*, *∆argB*, *adeA^−^*) (Supporting Information 1, Tables S1 and S2) [26], which has been widely used for biosynthesis of fungi-derived natural products due to its genetic tractability [27]. The resulted transformant was cultivated in the modified Czapek–Dox medium for four days, and then mycelia were harvested and extracted for analysis. Upon gas chromatography-mass spectrometry (GC–MS) analysis, we observed an additional peak at *m*/*z* = 272 [M]^+^ from the *tadA* harboring transformant, implying that TadA can catalyze the formation of a diterpene hydrocarbon (Supporting Information 1, Figure S1). The crude extract was also subjected to high-performance liquid chromatography–mass spectrometry (HPLC–MS) analysis. It showed that introduction of *tadA* led to the generation of two products **1** and **2**, but at low yields (Figure 3A, lines i and ii). To facilitate the isolation of these compounds, we co-expressed *tadA* with the GGPP synthase (GGPPS) gene (Supporting Information File 1, Note S1) derived from *Nodulisporium* sp. (No. 65-12-7-1) [28] in *A. oryzae* NSAR1. Although GGPP was not detected, production of **1** was increased by five-fold (Figure 3A, line iii). Finally, we isolated **1** and **2** through large-scale fermentation.

**Scheme 1:**
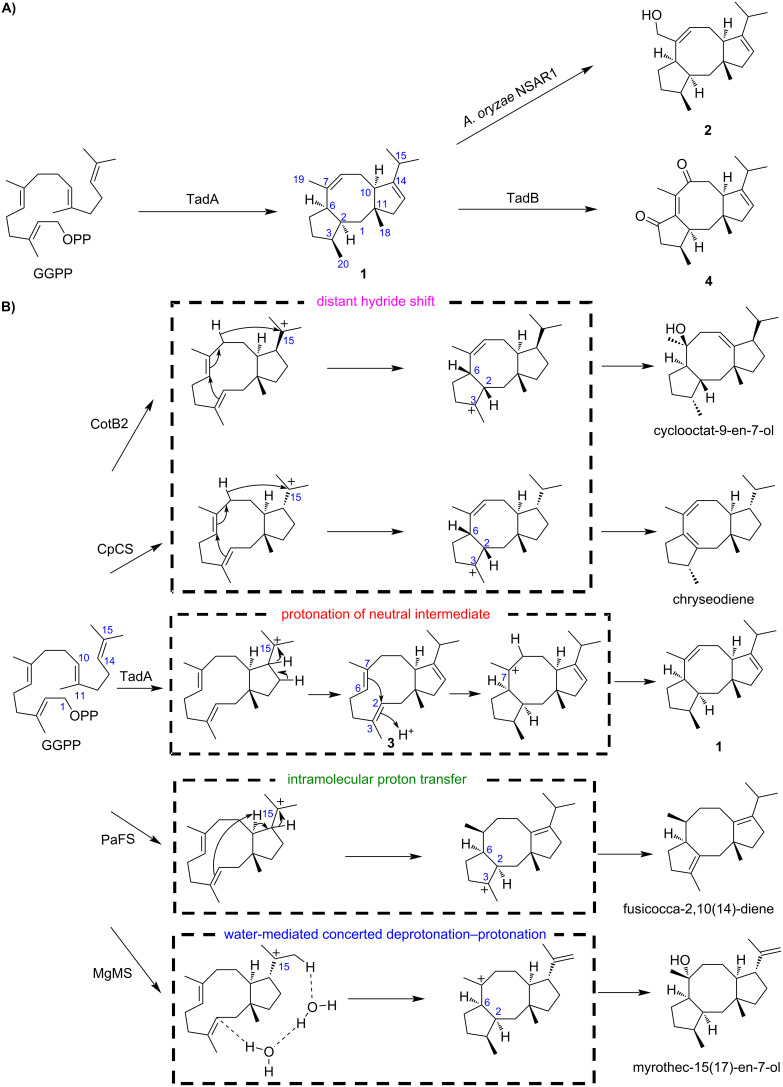
Biosynthesis of FC-type diterpenoids. A) The biosynthetic pathway of **1**, **2** and **4**. B) Cyclization mechanisms of **1** and reported FC-type diterpenes.

On the basis of the quasi-molecular ion at *m*/*z* 273.2599 [M + H]^+^ (calcd. for C_20_H_33_, 273.2582) (Supporting Information File 1, Figure S2) detected by high-resolution electrospray ionization mass spectrometry (HRESIMS), the molecular formula of **1** was deduced as C_20_H_32_, indicating that **1** has five degrees of unsaturation. The ^13^C NMR spectrum showed that there are four olefinic carbons (δ_C_ 153.2, 137.4, 125.1, 118.6) in **1**. We thus reasoned that **1** features a tricyclic system. Subsequently, the extensive NMR analysis established the planar structure of **1**, and its relative configuration was partially assigned as 2S*,3S*,6R*,10R* by the NOESY spectrum (Supporting Information File 1, Table S3 and Figures S3–S8). As to the stereochemistry of C11, quantum chemical calculations of ^13^C NMR chemical shifts were performed, which enabled us to determine that C18 and H10 are located on opposite sides of the five-membered ring (Supporting Information File 1, Figures S9 and S10). The conclusion was in agreement with NOE correlations between H10 and Ha12, and between H_3_18 and Hb12 (Supporting Information File 1, Table S3). The absolute configuration of **1** was later determined based on its oxidized product **4** generated by TadB. Based on these results, TadA was experimentally determined as a new FC-type DTS, which catalyzes the formation of talaro-7,13-diene (**1**, Scheme 1A).

According to the HRESIMS spectrum and NMR analysis (Supporting Information File 1, Table S4 and Figures S11–S17), **2** was established as the C19-hydroxylated form of **1**. As during heterologous expression in *A. oryzae*, shunt products could sometimes be generated by endogenous enzymes [29–30], we performed feeding experiments to test whether **2** was generated by *A. oryzae* NSAR1. The result showed that the heterologous host indeed converts **1** to talaro-7,13-dien-19-ol (**2**, Scheme 1A; Supporting Information File 1, Figure S18).

### Mechanistic characterization of TadA

So far, cyclization mechanisms of FC-type diterpenes afforded by PaFS [18], MgMS [20], CotB2 [19], and CpCS [21] have been deciphered. All these enzymes undergo a common C1,11–C10,14-bicyclization to form a C15 carbocation, but differ a lot at the following C2,6 cyclization (Scheme 1B). CotB2 and CpCS trigger the C2,6 cyclization via a distant hydride shift, whereas PaFS employs an intramolecular proton transfer. We recently showed that a water-mediated concerted deprotonation–protonation is required for the MgMS-mediated cyclization [20]. In order to probe the mechanism underlying the cyclization of **1**, we used His_6_-tagged TadA to carry out in vitro enzymatic reactions with or without addition of deuterated water (D_2_O). GC–MS analysis showed that when the reaction mixture was supplemented with D_2_O, the ion peak at *m*/*z* 273 was observed, indicating that exogenous deuterium was incorporated into **1** (Supporting Information File 1, Figure S19). This suggests that TadA might adopt a similar strategy as MgMS to initiate C2,6 cyclization though protonation at C2 by bulky water.

To obtain further insights into the C2,6-cyclization process of TadA, its three-dimensional (3D) protein structure was constructed with SWISS-MODEL using PaFS (PDB entry 5er8) as the template, and the proposed bicyclic neutral intermediate was docked into the active pocket of TadA (Supporting Information File 1, Figure S20). We searched for the amino acid residues surrounding C2 or C3, which might be involved in the C2 protonation, and found the candidate residue Tyr91. In the corresponding site, PaFS possesses a histidine residue (Supporting Information File 1, Figure S20). To test its role, Tyr91 in TadA was mutated to His, and then analyzed by an in vitro enzymatic assay. The result showed that the variant could give an additional product **3** (Supporting Information File 1, Figure S21). For isolation of **3**, the mutated *tadA*, along with GGPPS gene, was introduced into *A. oryzae* NSAR1, and the resulted transformant produced **3** at a titer of 0.6 mg/L (Figure 3A, line iv). By comparison of NMR data and specific optical rotation values, together with quantum chemical calculations of ^13^C NMR chemical shifts, **3** was determined to be (3a*S*,5*E*,9*E*,12a*R*)-3,3a,4,7,8,11,12,12a-octahydro-3a,6,10-trimethyl-1-(1-methylethyl)cyclopentacycloundecene [31] (Supporting Information File 1, Figures S22–S24), indicating that Tyr91 is an essential amino acid residue involved in C2,6-cyclization. Intriguingly, through careful examination of the GC–MS profile of the transformant expressing *tadA*, we also observed the appearance of **3**, the content of which is rather lower than that in the *tadA^Y91H^*-containing transformant (Supporting Information File 1, Figure S25). Based on these, we propose that though both MgMS and TadA use protonation-induced C2,6 cyclization, TadA likely adopts a more asynchronous process to give a neutral intermediate **3** first followed by protonation to form **1**, which is different from the highly concerted deprotonation–protonation process employed by MgMS (Scheme 1B). Further isotope labeling experiments and density functional theory (DFT) calculations are needed so as to gain deeper insight into the cyclization mechanism of **1**.

### Functional analysis of the cytochrome P450 enzyme TadB

Due to the significance of tailoring enzymes in terms of structural diversification and bioactivity improvement [11], we then turned to the associated cytochrome P450 gene *tadB*. We found that introduction of *tadB* into the transformant possessing *tadA* and the GGPPS gene could result in disappearance of **1**, but no additional products were observed at 208 nm (Figure 3A, line v). When the detective wavelength was switched from 208 nm to 254 nm, additional product **4** was detected (Figure 3B, lines i and ii), which was elucidated to be the highly oxygenated product of **1** through the exhaustive NMR analysis (Supporting Information File 1, Table S5 and Figures S26–S32). Based on NMR data and the fact that **4** is derived from **1**, the relative configuration of **4** was assigned as 2S*,3S*,10R*,11R*. And as the experimental electronic circular dichroism (ECD) spectrum of **4** resembled the calculated ECD spectrum of (2*S*,3*S*,10*R*,11*R*)-**4**, the absolute configuration of **4** was determined to be 2*S*,3*S*,10*R*,11*R* (Supporting Information File 1, Figures S33 and S34). The highly oxidized properties of talaro-6,13-dien-5,8-dione (**4**) indicate that TadB is a multifunctional P450 enzyme (Scheme 1A). Since no other intermediates were obtained, we could not determine the exact order of TadB-mediated oxidation, or exclude the possibility that endogenous enzymes from *A. oryzae* NSAR1 was involved in formation of **4**.

In addition, elucidating the stereochemistry of **4** also allowed us to assign the absolute configurations of **1** and **2** as 2*S*,3*S*,6*R*,10*R*,11*R*, raising a possibility that **1** might be the biosynthetic precursor of roussoellol C, a cytotoxic FC-type diterpenoid isolated from *T. purpurogenus* PP-414 [7]. It will be important to elucidate the biosynthetic pathway of roussoellol C, providing enzymatic tools for expanding the chemical diversity of talaro-7,13-diene related FC-type diterpenoids via combinational biosynthesis [14].

## Conclusion

We have identified a new fungal FC-type DTS, which is responsible for the biosynthesis of talaro-7,13-diene (**1**). Further mechanistic studies revealed that 2,6-cyclization in the formation of **1** is likely to be triggered by protonation of the neutral intermediate **3**, and Tyr91 in TadA plays a significant role in this process. The associated P450 enzyme TadB can catalyze multiple oxidation of **1** to highly oxygenated product talaro-6,13-dien-5,8-dione (**4**). This study has expanded the enzyme inventory for structural diversification of FC-type diterpenoids.

## Supporting Information

File 1 Experimental methods, nucleotide sequence, tables, and figures.
