# Supplementary material for: Characterization of a new fusicoccane-type diterpene synthase and an associated P450 enzyme
Source: Beilstein J Org Chem. 2022 Oct 5;18:1396–402. doi: 10.3762/bjoc.18.144 (PMC9551204; doi:10.3762/bjoc.18.144)
Supplement: File 1 — Experimental methods, nucleotide sequence, tables, and figures. [file Beilstein_J_Org_Chem-18-1396-s001.pdf]

BEILSTEIN JOURNAL OF ORGANIC CHEMISTRY

Supporting Information

for

Characterization of a new fusicoccane-type diterpene synthase and an associated P450 enzyme

Jia-Hua Huang, Jian-Ming Lv, Liang-Yan Xiao, Qian Xu, Fu-Long Lin, Gao-Qian Wang, Guo-Dong Chen, Sheng-Ying Qin, Dan Hu and Hao Gao

Beilstein J. Org. Chem. **2022,** *18,* 1396–1402. doi:10.3762/bjoc.18.144

Experimental methods, nucleotide sequence, tables, and figures

License and Terms: This is a supporting information file under the terms of the Creative Commons Attribution License (https://creativecommons.org/licenses/by/4.0). Please note that the reuse, redistribution and reproduction in particular requires that the author(s) and source are credited and that individual graphics may be subject to special legal provisions.
The license is subject to the *Beilstein Journal of Organic Chemistry* terms and conditions: (https://www.beilstein-journals.org/bjoc/terms)

Table of Contents

Supplementary Methods S4
General materials and experimental procedures S4
Strains and media S5
Construction of heterologous expression plasmids S5
Transformation of *A. oryzae* NSAR1 .... S5
Fermentation of *A. oryzae* NSAR1 transformants S6
Heterologous expression of *tadA* and *tadA*^{Y91H} in *E. coli* Rosetta(DE3), and purification of TadA S7
Feeding experiments and in vitro enzymatic assay S7
Homology modeling and molecular docking S8
Analysis of metabolites S8
Purification procedure for compounds **1**–**4** S9
Quantum chemical calculations of ^{13}C NMR chemical shifts S10
Quantum chemical ECD calculations S10
Structural characterization S11
Supplementary Notes S12
Note S1. The nucleotide sequence of GGPPS from *Nodulisporium* sp. 65-12-7-1 S12
Supplementary Tables S13
Table S1. Primers used for constructing recombinant plasmids S13
Table S2. Plasmids used in the study S15
Table S3. NMR assignments for **1** in C_6D_6 (^{1}H at 400 MHz and ^{13}C at 100 MHz).. S16
Table S4. NMR assignments for **2** in $CDCl_3$ (^{1}H at 400 MHz and ^{13}C at 100 MHz) S17
Table S5. NMR assignments for **4** in $CDCl_3$ (^{1}H at 600 MHz and ^{13}C at 150 MHz) S18
Supplementary Figures S19
Figure S1. Functional analysis of TadA S19
Figure S2. HRESIMS spectrum of **1** S20
Figure S3. ^{1}H NMR spectrum of **1** in C_6D_6 at 400 MHz S20
Figure S4. ^{13}C NMR spectrum of **1** in C_6D_6 at 100 MHz S21
Figure S5. ^{1}H-^{1}H COSY spectrum of 1 in C_6D_6 at 400 MHz S21
Figure S6. HSQC spectrum of **1** in C_6D_6 at 400 MHz S22
Figure S7. HMBC spectrum of **1** in C_6D_6 at 400 MHz S22
Figure S8. NOESY spectrum of **1** in C_6D_6 at 600 MHz S23
Figure S9. Most stable conformers of (2*S**,3*S**,6*R**,10*R**,11*S**)-**1A** and (2*S**, 3*S**, 6*R**, 10*R**, 11*R**)-**1B** S24
Figure S10. Determination of the relative configuration of C11 in **1** on the basis of R^2, mean absolute error, and DP4+ probability S25
Figure S11. HRESIMS spectrum of **2** S26
Figure S12. ^{1}H NMR spectrum of **2** in $CDCl_3$ at 400 MHz S26
Figure S13. ^{13}C NMR spectrum of **2** in $CDCl_3$ at 100 MHz S27
Figure S14. ^{1}H-^{1}H COSY spectrum of **2** in $CDCl_3$ at 400 MHz S27
Figure S15. HSQC spectrum of **2** in $CDCl_3$ at 400 MHz S28

Figure S16. HMBC spectrum of **2** in $CDCl_3$ at 600 MHz S28
Figure S17. NOESY spectrum of **2** in $CDCl_3$ at 600 MHz S29
Figure S18. Extracted ion chromatogram (EIC) of metabolites of *A. oryzae* NSAR1 fed with **1** or DMSO S30
Figure S19. In vitro assay of TadA with addition of D_2O or H_2O S31
Figure S20. Identification of key amino acid residues for the protonation of the putative neutral intermediate S32
Figure S21. Functional analysis of $TadA^{Y91H}$ S32
Figure S22. ^{13}C NMR spectrum of **3** in $CDCl_3$ at 100 MHz S33
Figure S23. Most stable conformers of (10*R**,11*S**)-**3A** and (10*R**,11*R**)-**3B** S34
Figure S24. Determination of the relative configuration of **3** on the basis of R^2, mean absolute error, and DP4+ probability. S35
Figure S25. GC–MS analysis of metabolites from *A. oryzae* NSAR1 transformants expressing TadA or $TadA^{Y91H}$ S36
Figure S26. HRESIMS spectrum of **4** S37
Figure S27. ^{1}H NMR spectrum of **4** in $CDCl_3$ at 600 MHz S37
Figure S28. ^{13}C NMR spectrum of **4** in $CDCl_3$ at 150 MHz S38
Figure S29. ^{1}H-^{1}H COSY spectrum of **4** in $CDCl_3$ at 600 MHz S38
Figure S30. HSQC spectrum of **4** in $CDCl_3$ at 600 MHz S39
Figure S31. HMBC spectrum of **4** in $CDCl_3$ at 600 MHz S39
Figure S32. NOESY spectrum of **4** in $CDCl_3$ at 600 MHz S40
Figure S33. Most stable conformers of (2*S*,3*S*,10*R*,11*R*)-**4** S41
Figure S34. Comparison of experimental ECD spectrum of **4** and calculated ECD spectra of (2*S*,3*S*,10*R*,11*R*)-4/(2*R*,3*R*,10*S*,11*S*)-**4** S42
Supplementary References S43

Supplementary Methods

General materials and experimental procedures

Acetonitrile (CH_3CN) was obtained from Oceanpak Alexative Chemical Co., Ltd. (Gothenburg, Sweden). Methanol (CH_3OH) was purchased from Yuwang Industrial Co., Ltd. (Yucheng, China). The other chemicals were purchased from HuaDa Chemical Co., Ltd. (Guangzhou, China). The biochemical reagents and kits used in this work were purchased from TaKaRa Bio Inc. (Dalian, China), Tsingke Biotech Co., Ltd. (Beijing, China), or Sangon Biotech Co., Ltd. (Shanghai, China), unless noted otherwise. The primer synthesis was performed by Tsingke Biotech Co., Ltd. (Beijing, China). PCR was carried out using a Mastercycler nexus gradient (Eppendorf, Hamburg, Germany).

HPLC-MS analysis was performed using a Dionex UltiMate 3000 HPLC system (Thermo Scientific, USA) with a COSMOSIL-Pack column (4.6 mm i.d. × 150 mm) and an amaZon SL ion trap mass spectrometer coupled with an atmospheric pressure chemical ionization (APCI) source (Bruker, USA). GC–MS analysis was performed on Agilent Technologies 7890B GC System coupled with 5977B MSD using an HP-5MS 30 Meter column (0.32 mm i.d., 0.25 μm film thickness). Column chromatography was carried out with silica gel (200–300 mesh) (Qingdao Haiyang Chemical Group Corporation, Qingdao, China). The semi-preparative HPLC was performed on a Dionex UltiMate 3000 HPLC system with a YMC-Pack ODS-A column (10.0 mm i.d. × 250 mm, 5 μm).

UV data, IR data, and optical rotations were, respectively, measured on a JASCO V-550 UV/vis spectrometer, JASCO FT/IR-4600 plus spectrometer, and JASCO P2000 digital polarimeter from JASCO International Co., Ltd. (Tokyo, Japan). ECD spectra were recorded in CH_3OH using a JASCO J-810 spectrophotometer (JASCO International Co., Ltd., Tokyo, Japan) at room temperature. The HRESIMS data were obtained from SYNAPT G2 high definition mass spectrometer (Waters, USA). 1D and 2D NMR spectra were recorded with the Bruker AV 400/600 spectrometers

(Faellanden, Switzerland) using the solvent signals ($CDCl_3$: δ_H 7.26/δ_C 77.0; C_6D_6: δ_H 7.16/δ_C 128.1) as the reference.

Strains and media

Talaromyces wortmannii ATCC 26942 was purchased from the American Type Culture Collection (ATCC), and was cultured in potato dextrose broth for DNA extraction. *Aspergillus oryzae* NSAR1 (*niaD*$^-$, *sC*$^-$, Δ*argB*, *adeA*$^-$)[1] was used as the host for heterologous expression in the modified Czapek-Dox (CD) medium (0.3% $NaNO_3$, 0.2% KCl, 0.05% $MgSO_4 \cdot 7H_2O$, 0.1% KH_2PO_4, 0.002% $FeSO_4 \cdot 7H_2O$, 1% polypeptone, 2% starch, pH 5.5). *Escherichia coli* DH5α (TaKaRa) was used for construction of recombinant plasmids, and *E. coli* Rosetta(DE3) (TaKaRa) was used for protein expression.

Construction of heterologous expression plasmids

tadA and *tadB* were amplified from the genomic DNA of *T. wortmannii* ATCC 26942. GGPP synthase gene was amplified from the genomic DNA of *Nodulisporium* sp. (No. 65-12-7-1)[2]. The resulted genes were inserted into the linearized pTAex3 vector to yield pTAex3-*tadA*, pTAex3-*GGPPS,* and pTAex3-*tadB*, respectively. Subsequently, the *tadA* and *GGPPS* expression cassettes, both of which contained the *amyB* promoter and terminator, were amplified from the pTAex3 based plasmids, respectively, and then were cloned into the SmaI digested pTAex3 vector to afford pTAex3-*tadA*-*GGPPS*. In the same way, the *tadB* expression cassette was ligated with the XbaI digested pAdeA vector to yield pAdeA-*tadB*. The mutated gene encoding $TadA^{Y91H}$ was constructed through amplification of *tadA* using mutagenesis primers, and then introduced into pTAex3. All the primers and plasmids used in this work are listed in Tables S1 and S2.

Transformation of *A. oryzae* NSAR1

The *A. oryzae* NSAR1 transformants were obtained via PEG-mediated protoplast

transformation. The spore suspension of *A. oryzae* NASR1 was inoculated in 10 mL DPY medium (2% dextrin, 1% polypeptone, 0.5% yeast extract, 0.05% $MgSO_4 \cdot 7H_2O$, 0.5% KH_2PO_4), and cultivated at 28 °C and 220 rpm for 2 days. Then the culture broth was transferred into 100 mL DPY. After growing for 1 day, mycelia were harvested by filtration, and digested using the Yatalase (Takara, Japan) solution (1% Yatalase, 0.6 M $(NH4)_2SO_4$, 50 mM maleic acid, pH 5.5) to remove the cell walls at 30 °C for 3 hours. The resulted protoplasts were washed with Solution 2 (1.2 M sorbitol, 50 mM $CaCl_2 \cdot 2H_2O$, 35 mM NaCl, 10 mM Tris-HCl, pH 7.5), and then adjusted to around 1.0 × 10^7 cell/mL with Solution 2. 10 μg plasmids (≈10 μL) and 200 μL protoplast suspension were gently mixed and placed on ice for 30 min. Subsequently, a total of 1.35 mL PEG solution (60% PEG4000, 50 mM $CaCl_2 \cdot 2H_2O$, 10 mM Tris-HCl, pH 7.5) was added in three times. After incubation at room temperature for 20 min, 5 mL Solution 2 was added, and the mixture was subjected to centrifugation at 1500 rpm for 10 min. The supernatant was discarded, and the protoplast pellet was resuspended in 200 μL Solution 2. Finally, the mixture was spread on the lower selective medium, which was then covered with the top selective medium. The selective medium contains 0.2% NH_4Cl, 0.1% $(NH_4)_2SO_4$, 0.05% KCl, 0.05% NaCl, 0.1% KH_2PO_4, 0.05% $MgSO_4 \cdot 7H_2O$, 0.002% $FeSO_4 \cdot 7H_2O$, 2% glucose, 1.2 M sorbitol and 1.5% agar (lower medium)/0.8% agar (top medium) as well as appropriate ingredients for auxotrophy-complementing. The plate was placed at 30 °C for 3–5 days, and the transformants would be obtained.

Fermentation of *A. oryzae* NSAR1 transformants

Mycelia of the *A. oryzae* transformant were inoculated into 10 mL DPY and cultivated at 28 ° C and 220 rpm for 2 days as the seed broth. Then the broth was transferred into 100 mL modified CD medium for induction of genes. After growing at 28 °C and 220 rpm for 5 days, mycelia were harvested and extracted with acetone. The extract was concentrated under reduced pressure, and then partitioned with hexane–water (1/1, v/v) for GC–MS analysis, or resuspended in methanol for HPLC–

MS analysis.

Heterologous expression of *tadA* and *tadA*^{Y91H} in *E. coli* Rosetta(DE3), and purification of TadA

The intronless *tadA* gene was amplified from the cDNA of the *A. oryzae* transformant harboring pTAex3-*tadA*. Then, the fragment was ligated with the pET28-MBP vector to yield pET28-MBP-TadA. As aforementioned, the intronless *tadA* variant was constructed, and then ligated with pET28-MBP to afford pET28-MBP-TadA^{Y91H}. The recombinant plasmid was then introduced into *E. coli* Rosetta(DE3) according to the manufacture′s instruction. 1 mL preculture broth was inoculated into 100 mL LB medium with kanamycin (50 g/mL), and cultivated at 37 °C and 220 rpm until OD_{600} reached 0.4–0.6. Subsequently, expression of the exogeneous gene was induced with addition of 0.2 mM isopropyl β-D-1-thiogalactopyranoside (IPTG), and the mixture was further cultivated at 18 °C for 12–16 h.

In order to purify the recombinant TadA, the cells from 1 L culture broth were harvested by centrifugation, and then resuspended in 40 mL lysis buffer (50 mM Tris-HCl, 250 mM NaCl, 5 mM $MgCl_2$, 5 mM imidazole, 10% glycerol, pH 8.0). The mixture was subjected to ultrasonication-assisted lysis, and then centrifuged at 5800 g for 20 min. The soluble fraction was loaded onto a column containing 6 mL Ni^{2+}-NTA agarose, and the native proteins were eluted with 300 mL wash solution (50 mM Tris-HCl, 250 mM NaCl, 5 mM $MgCl_2$, 20 mM imidazole, 10% glycerol, pH 8.0). Finally, His_6-tagged recombinant TadA was eluted with 20 mL elution buffer (50 mM Tris-HCl, 250 mM NaCl, 5 mM $MgCl_2$, 200 mM imidazole, 10% glycerol, pH 8.0). After desalted and concentrated, the resulted protein was subjected to SDS-PAGE analysis for purity evaluation. And the protein concentration was measured with an Eppendorf BioPhotometer D30.

Feeding experiments and in vitro enzymatic assay

For feeding experiments, *A. oryzae* NSAR1 was cultured in 10 mL DPY medium for

2 days, and then inoculated into 100 mL modified CD medium. After growing for 18 h, the culture broth was supplemented with 5.0 mg of compound **1** (dissolved in 100 μL DMSO), and further incubated for 5 days. Mycelia were extracted for HPLC–MS analysis.

For in vitro enzymatic assay using soluble fraction of *E. coli* Rosetta(DE3) transformants harboring *tadA* or its variant, the cells from 100 mL culture broth were harvested, and then resuspended in 4 mL lysis buffer (50 mM Tris-HCl, 5 mM $MgCl_2$, 2.0 mM DTT, pH 8.0) for ultrasonication assisted lysis. After centrifugation, 300 μL supernatant and 15 μL of 1 mg/mL GGPP were mixed together, and incubated at 30 °C for 4 h. Subsequently, the reaction mixture was extracted with EtOAc. After removal of solvent under reduced pressure, the crude extract was dissolved in *n*-hexane for GC–MS analysis.

For in vitro enzymatic assay using the purified recombinant TadA, a total volume of 300 μL reaction mixture, containing 20 μL of 171.5 μM TadA, 15 μL of 1 mg/mL GGPP, 15 μL of 1 M Tris-HCl (pH 8.0), 15 μL of 100 mM $MgCl_2$, 6 μL of 100 mM DTT, and 229 μL of D_2O or H_2O, was incubated at 30 °C for 4 h. Subsequently, the reaction mixture was extracted with EtOAc. After removal of solvent under reduced pressure, the crude extract was dissolved in *n*-hexane for GC–MS analysis.

Homology modeling and molecular docking

The 3D structure of TadA was built by the SWISS-MODEL web server (swissmodel.expasy.org) using the crystal structure of PaFS (5er8) as the template. Molecular docking of compound **3** into the active pocket of TadA was performed by AutoDock Vina using standard parameters[3].

Analysis of metabolites

For HPLC–MS analysis, the mobile phase was composed of water with 0.1% formic acid (A) and acetonitrile with 0.1% formic acid (B), and the sample was subjected to a linear gradient elution of 50–100% B (0–25 min) and 100% B (25–60 min) with the flow

rate of 1 mL/min.

For GC–MS analysis, the ionization chamber temperature was set to 230 °C, and the electron impact ionization voltage was set to 70 eV. The oven temperature was initially kept at 50 °C for 3 min, then increased to 70 °C at a rate of 20 °C/min, and held for 1 min. Subsequently, the oven temperature underwent a second ramp up of 15 °C/min to 300 °C, followed by a hold for 3 min. Helium was used as carrier gas at the rate of 1 mL/min.

Purification procedure for compounds 1–4

Mycelia from 5 L culture of the *A. oryzae* NSAR1 transformant harboring *tadA* and GGPPS gene were extracted with acetone at room temperature. Then the extract (1.5 g) was subjected to silica gel column chromatography with cyclohexane. Fraction containing **1** was further purified by semi-preparative HPLC (YMC-Pack ODS-A column, 3 mL/min) with isocratic elution of 92% CH_3OH–THF to yield **1** (t_R: 36.5 min, 25.0 mg). Fraction containing **2** was further purified by the same semi-preparative HPLC with isocratic elution of 85% CH_3CN–H_2O containing 0.1% formic acid to yield **2** (t_R: 25.5 min, 5.0 mg).

Mycelia from 6 L culture of the *A. oryzae* NSAR1 transformant harboring *tadA*^{Y91H} and GGPPS gene were extracted with acetone at room temperature. Then the extract (2.4 g) was subjected to silica gel column chromatography. Fraction containing **3** was further purified by semi-preparative HPLC (YMC-Pack ODS-A column, 3 mL/min) with isocratic elution of 90% CH_3OH–THF to yield **3** (t_R: 33.0 min, 3.6 mg).

Mycelia from 18 L culture of the *A. oryzae* NSAR1 transformant harboring *tadA*, GGPPS gene, and *tadB* were extracted with acetone at room temperature. Then the extract (6.5 g) was subjected to silica gel column chromatography. Fraction containing **4** was further purified by semi-preparative HPLC (YMC-Pack ODS-A column, 3 mL/min) with isocratic elution of 85% CH_3CN–H_2O containing 0.1% formic acid to yield **4** (t_R: 19.7 min, 5.0 mg).

Quantum chemical calculations of ^{13}C NMR chemical shifts

The molecules of (2*S**,3*S**,6*R**,10*R**,11*S**)-**1A**/(2*S**,3*S**,6*R**,10*R**,11*R**)-**1B** or (10*R**,11*S**)-**3A**/(10*R**,11*R**)-**3B** were converted into SMILES codes before their initial 3D structures were generated with CORINA version 3.4. Conformer databases were generated in CONFLEX version 7.0 by using the MMFF94s force-field with an energy window for acceptable conformers (ewindow) of 5 kcal/mol above the ground state, a maximum number of conformations per molecule (maxconfs) of 100, and an RMSD cutoff (rmsd) of 0.5 Å. Then each acceptable conformer was optimized with HF/6-31G(d) method in Gaussian09[4]. Further optimization at the wB97XD/6-31G(d) level determined the dihedral angles. From this, most stable conformers were determined. The optimized conformers were used for ^{13}C NMR calculations, which were performed with Gaussian09 (mPW1PW91/6-31G(d)). The solvent effects were taken into account by the polarizable-conductor calculation model (PCM, C_6D_6 or $CDCl_3$ as the solvent). The comparison was judged by R square (R^2) analysis, mean absolute error (MAE), and DP4+ probability[5].

Quantum chemical ECD calculations

The molecules of (2*S*,3*S*,10*R*,11*R*)-**4** and (2*R*,3*R*,10*S*,11*S*)-**4** were converted into SMILES codes before their initial 3D structures were generated with CORINA version 3.4. Conformer databases were generated in CONFLEX version 7.0 using the MMFF94s force-field, with an energy window for acceptable conformers (ewindow) of 5 kcal/mol above the ground state, a maximum number of conformations per molecule (maxconfs) of 100, and an RMSD cutoff (rmsd) of 0.5 Å. Then each acceptable conformer was optimized with HF/6-31G(d) method in Gaussian09[4]. Further optimization at the wB97XD/6-31G(d) level determined the dihedral angles. From this, five most stable conformers were found. The optimized conformers were used for the ECD calculations, which were performed with Gaussian09 (wB97XD/TZVP). The solvent effects were taken into account by the polarizable-conductor calculation model (IEFPCM, methanol as the solvent). Comparison of the experimental and calculated

spectra were carried out with the software SpecDis[6]. It was also used to perform a UV shift to the ECD spectra, Gaussian broadening of the excitations, and Boltzmann weighting of the spectra.

Structural characterization

Talaro-7,13-diene (**1**): colorless oil; $[\alpha]_D^{32}$ = -10.6 (*c* 1.0, $CHCl_3$); IR (KBr) ν_{max} 2959, 2927, 2845, 1455, 1437, 1379, 1362, 809 cm^{-1}; HRESIMS (positive) *m/z* 273.2599 $[M + H]^+$ (calcd for $C_{20}H_{33}$, 273.2582), see Fig. S2; NMR spectra, see Figs. S3-S8; NMR data, see Table S3.

Talaro-7,13-dien-19-ol (**2**): colorless oil; $[\alpha]_D^{32}$ = -40.0 (*c* 0.5, $CHCl_3$); IR (KBr) ν_{max} 3054, 2962, 2880, 1453, 1376, 1045 cm^{-1}; HRESIMS (positive) *m/z* 289.2543 $[M + H]^+$ (calcd. for $C_{20}H_{33}O$, 289.2531), see Fig. S11; NMR spectra, see Figs. S12-S17; NMR data, see Table S4.

3: colorless oil; $[\alpha]_D^{27}$ = +10.8 (*c* 0.2, $CHCl_3$); NMR spectra, see Fig. S22; ^{13}C NMR (100 MHz, $CDCl_3$) δ_C 154.3, 134.4, 133.2, 128.7, 125.4, 118.4, 48.4, 47.0, 46.1, 41.2, 40.0, 38.1, 27.1, 25.9, 24.4, 23.3, 22.4, 22.2, 16.0, 15.1; The NMR data are in good agreement with those of (3a*S*,5*E*,9*E*,12a*R*)-3,3a,4,7,8,11,12,12a-octahydro-3a,6,10-trimethyl-1-(1-methylethyl)cyclopentacycloundecene (CAS Registry Number: 1404310-43-5)[7].

Talaro-6,13-dien-5,8-dione (**4**): colorless oil; $[\alpha]_D^{31}$ = +9.9 (*c* 1.0, $CHCl_3$); UV ($CHCl_3$) λ_{max} (log *ε*) 244 (2.09) nm; IR (KBr) ν_{max} 2955, 2931, 2876, 1713, 1626, 1469, 1408, 1375 cm^{-1}; ECD (c 49 μM, CH_3OH) λ_{max} ($\Delta\varepsilon$) 241.5 (-10.24), 310.5 (+4.95) nm; HRESIMS (positive) *m/z* 301.2162 $[M + H]^+$ (calcd. for $C_{20}H_{29}O_2$, 301.2168), see Fig. S26; NMR spectra, see Figs. S27-S32; NMR data, see Table S5.

Supplementary Notes

Note S1. The nucleotide sequence of GGPPS from *Nodulisporium* sp. 65-12-7-1

ATGGCCTCCAACGCCAATTCCAGCAATATATCTCTCCAGTCGCCCAACGCCATT
CCTCCCAGGACCTCCTCGACGGGCTACATTGCTCCTTTCCCTCCCGCAAAATCT
GTCCTGCGGCCTGTTCCAGAGTCGGACTGGCTCGGTCAGAATAACGGAAGACA
TCGAAGCACCTCAACTACCGCTATACCCTTGACCGGCATGCATGCCACGGGGC
CCCAGGACCCCGCGCGGTACGAGACCGAAGACCTGAACTATACCTCGAGAAAG
ACATGGTCTGAGCAGAAGGAAAAGGTCCTCGTGGGCCCTTTTGAGTACTTGTAC
GCTCAGCCCGGGAAAGACTTCCGCACGCTCATGGTCAATTCCTTCAATGCGTG
GCTTGAAGTCCCCCAGGAAAGCTTGGACGTCATCACAAGGGTTGTCGGCATGC
TACACACGGCATCGTTACTCATTGATGATGTTGAGGACAATTCATCCCTCCGGA
GGGGTTTACCTGTCGCTCATAACATTTTTGGCACGGCACAAACTATAAACTCGG
CCAACTACATCTACTTCTGTGCCTTGCAGGAGTTACAAAAGCTAAAGAACCCAAA
GGCGATCAATGTCTACACGGAAGAATTGCTGAACCTCCACCGCGGTCAAGGCA
TGGATCTCTTCTGGCGGGACACTCTAACGTGCCCCACCGAAGAGGAGTACCTG
GAAATGGTCGGCAACAAAACTGGGGGTCTCTTCCGCTTGGCCATCAAGCTGAT
GCAGGCCGAGTCAGGCACCCTCATCGACTGCGTGCCCTTGGTCAACATACTAG
GCATAATCTTCCAGATCCAAGACGACTACCGGAACCTGTTGAGCCCCGAGTACG
GCCATAACAAAGGCCTTTGCGAAGACTTGACAGAGGGCAAATTCTCATTCCTTA
TCATCCACAGCATACGTTCAAACCCTTCAAATCTCCAGCTTCTCAATATTCTCAA
GCAGAAGACCAAAGATGACGAGGTCAAAAGATATGCCGTCAAGTATATGGAGA
GCACGGGAAGCTTCGAGTACAGTCAAAAAGTCATTGCCGTCTTAGTCGAGAGA
GCGAGAAAGATGGCGGATGAGCTGGATGCTGGGAGGGGGAAGAGCGTCGGTA
TTCATAAAATCTTGGACAAGCTGGTGGTATAA

Supplementary Tables

Table S1. Primers used for constructing recombinant plasmids.

**Primer**	**Sequence (5′ to 3′)**	**Usage**
tadA-EcoRI-F	CCGGAATTCAGACAAAATGGACTTCAAAT	Cloning of *tadA* from *T. wortmannii* ATCC 26942 genome
tadA-KpnI-R	CGGGGTACCATTCCTTTAAGCTAAGCTAT	
Inf-*tadB*-F	TCGAGCTCGGTACCCTTTCAGCTATGAATTTCGCC	Cloning of *tadB* from *T. wortmannii* ATCC 26942 genome
Inf-*tadB*-R	CTACTACAGATCCCCAGCTACTTGATCATCACTCT	
inf-*GGPPS*-F	TCGAGCTCGGTACCCGTGGCAACCATGGCCTCCAA	Cloning of GGPPS gene from *Nodulisporium* sp. (No. 65-12-7-1) genome
inf-*GGPPS*-R	CTACTACAGATCCCCCTGCCCACCGGATCATATTG	
Inf -*GGPPS*-pTAex3-Parm-F	TCGAGCTCGGTACCCCACAGCAAGCTC	Construction of recombinant pTAex3 plasmid containing *tadA* and GGPPS gene
Inf -pTAex3-Tamy-R_1	AACGCGCTCGCGAGCAAGTACCATACAGTACCGCG	
Inf -pTAex3-Parm-F_1	GCTCGCGAGCGCGTTCCACTGCATCATCAGTCTAG	
Inf-*GGPPS*-pTAex3-Tamy-R	CTACTACAGATCCCCCTGC	
Inf –pAdeA-Parm-F	GCAGGTCGACTCTAGACGACTCCAATCTTCAAGAGC	Construction of recombinant pAdeA plasmid containing *tadB*
Inf -pAdeA-Tamy-R	TAGTAGATCCTCTAGAGTAAGATACATGAGCTTCGG	
PT-Mutant-Y91H--F	CCAACGAGTTTGCATTTCTACATGATGGTATAAACGTTGAAAG	Construction of *tadA*^{Y91H} with Inf -*GGPPS*-

mutant-Y91H-R	TAGAAATGCAAACTCGTTGG	pTAex3-Parm-F and Inf -pTAex3-Tamy-R_1
Inf-mbp-NdeI-TadA-F	ACTTCCAGTCACATATGATGGACTTCAAATATTCTAAAA	Cloning of the intron-free *tadA* from the cDNA of *A. oryzae* transformant harboring pTAex3-*tadA*
Inf-mbp-HindIII-TadA-R	GTGCGGCCGCAAGCTTTAAAATCTGCTCATATACTCC	
mutant-Y91H-F	CCAACGAGTTTGCATTTCTACATGATGATAAGACTGAGGATTT	Construction of intron-free *tadA*^{Y91H} with Inf-mbp-NdeI-TadA-F and Inf-mbp-HindIII-TadA-R
mutant-Y91H-R	TAGAAATGCAAACTCGTTGG	

Table S2. Plasmids used in the study.

Plasmid	Characteristic	Source
pTAex3	Plasmid containing *argB* maker gene cassette for gene expression in *A. oryzae* NSAR1 along with the ampicillin resistance gene cassette	Fujii *et al.*[8]
pAdeA	Plasmid containing *adeA* maker gene cassette for gene expression in *A. oryzae* NSAR1 along with the ampicillin resistance gene cassette	Jin *et al.*[9]
pTAex3-*tadA*	pTAex3 containing *tadA* under the *amyB* promoter	This work
pTAex3-*tadB*	pTAex3 containing *tadB* under the *amyB* promoter	This work
pTAex3-*GGPPS*	pTAex3 containing GGPPS gene under the *amyB* promoter	This work
pTAex3-*tadA-GGPPS*	pTAex3 containing *tadA* and GGPPS gene under the *amyB* promoter	This work
pTAex3-*tadA*^{Y91H}-*GGPPS*	pTAex3 containing *tadA*^{Y91H} and GGPPS gene under the *amyB* promoter	This work
pAdeA-*tadB*	pAdeA containing *tadB* under the *amyB* promoter	This work
pET28-MBP	pET28a containing MBP gene under the T7 promoter along with the kanamycin resistance gene cassette	Lin *et al.*[10]
pET28-MBP-TadA	pET28a-MBP containing intronless *tadA* under the T7 promoter	This work
pET28-MBP-TadA^{Y91H}	pET28a-MBP containing intronless *tadA*^{Y91H} under the T7 promoter	This work

Table S3. NMR assignments for 1 in C_6D_6 (^1H at 400 MHz and ^{13}C at 100 MHz).

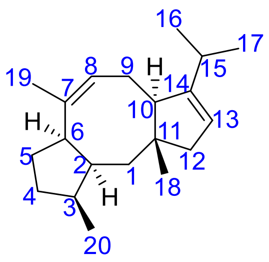

No.	δ_C, type	δ_H (J in Hz)[a]	^1H–^1H COSY	HMBC	NOESY[b]
1	38.0, CH_2	a: 1.48	1b, 2	2, 3, 6, 10, 11, 12	12a
		b: 1.34, t (13.0)	1a, 2	2, 3, 6, 11, 12, 18	
2	46.7, CH	2.34	1a, 1b, 3, 6	1, 3, 6, 7, 11, 20	4a, 10
3	38.2, CH	1.93	2, 4a, 4b, 20		
4	34.6, CH_2	a: 1.52	3, 4b, 5a, 5b	2, 3, 6, 20	2, 6
		b: 1.41	3, 4a, 5a, 5b	2, 3, 6, 20	
5	27.5, CH_2	a: 1.84	4a, 4b, 5b, 6	2, 4, 6, 7	
		b: 1.61	4a, 4b, 5a, 6	3, 4	
6	42.5, CH	3.26, q (9.2)	2, 5a, 5b	1, 2, 3, 5, 7, 8, 19	4a, 9a, 10
7	137.4, C				
8	125.1, CH	5.60, br d (8.3)	9a, 9b, 19	6, 9, 10, 19	18, 19
9	26.7, CH_2	a: 2.51	8, 9b, 10	8, 11	6, 15, 16
		b: 1.90	8, 9a, 10	7, 8, 10, 11, 14	15
10	49.3, CH	3.36	9a, 9b, 12a, 12b, 13	14	2, 6, 12a, 16
11	46.7, C				
12	46.2, CH_2	a: 2.23	10, 12b, 13	13, 14, 18	1a, 10
		b: 1.84	10, 12a, 13	10, 11, 13, 14, 18	18
13	118.6, CH	5.30, br s	10, 12a, 12b	10, 11, 12, 14, 15	17
14	153.2, C				
15	27.6, CH	2.19	16, 17	13, 14, 16	9a, 9b
16	22.8, CH_3	1.02, d (6.8)	15	14, 15, 17	9a, 10
17	21.3, CH_3	1.10, d (6.8)	15	14, 15, 16	13
18	25.1, CH_3	1.05, s		1, 10, 11, 12	8, 12b
19	24.2, CH_3	1.73, br s	8	6, 7, 8	8, 20
20	15.5, CH_3	0.75, d (7.2)	3	2, 3, 4	19

[a] The indiscernible signals from overlap or the complex multiplicity are reported without designating multiplicity.

[b] The data were measured at 600 MHz.

Table S4. NMR assignments for 2 in $CDCl_3$ (^1H at 400 MHz and ^{13}C at 100 MHz).

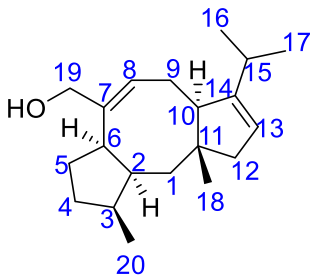

No.	δ_C, type	δ_H (J in Hz)[a]	^1H–^1H COSY	HMBC[b]	NOESY[b]
1	38.0, CH_2	a: 1.48	1b, 2	2, 3, 6, 10, 11, 12	12a, 20
		b: 1.21, t (13.3)	1a, 2	2, 6, 11, 12, 18	18, 20
2	46.2, CH	2.45	1a, 1b, 3, 6	1, 3, 6, 7, 11, 20	4a, 10, 12a
3	38.1, CH	2.05	2, 4a, 4b, 20	2, 4, 5, 6, 20	
4	34.1, CH_2	a: 1.65	3, 4b, 5a, 5b	3, 5, 20	2, 6
		b: 1.52	3, 4a, 5a, 5b	2, 5, 6, 20	
5	26.9, CH_2	a: 1.96	4a, 4b, 5b, 6	4, 6, 7	19a, 19b, 20
		b: 1.81	4a, 4b, 5a, 6	2, 3, 4, 6	
6	41.1, CH	3.31	2, 5a, 5b	1, 2, 3, 5, 7, 8, 19	4a
7	140.4, C				
8	128.8, CH	5.86, br d (7.9)	9a, 9b	6, 7, 9, 10, 19	18, 19a, 19b
9	26.4, CH_2	a: 2.48	8, 9b, 10	7, 8, 10, 11	15, 16
		b: 1.96	8, 9a, 10	7, 8, 10, 14	18
10	48.4, CH	3.25	9a, 9b, 13	13, 14, 18	2, 12a, 16
11	46.2, C				
12	45.7, CH_2	a: 2.14	12b, 13	13, 14, 18	1a, 2, 10
		b: 1.74	12a, 13	10, 11, 13, 14, 18	18
13	118.1 CH	5.19, br s	10, 12a, 12b	10, 14	17
14	152.5, C				
15	27.2, CH	2.18	16, 17	13, 14, 16, 17	9a
16	22.5, CH_3	0.98, d (6.6)	15	14, 15, 17	9a, 10
17	21.0, CH_3	1.04, d (6.6)	15	14, 15, 16	13
18	24.7, CH_3	0.85, s		1, 10, 11, 12	1b, 8, 9b, 12b
19	67.9, CH_2	a: 4.12, d (12.3)	19b	6, 7, 8	5a, 8, 20
		b: 4.02, d (12.3)	19a	6, 7, 8	5a, 8, 20
20	15.0, CH_3	0.76, d (7.2)	3	2, 3, 4	1a, 1b, 5a, 19a, 19b

[a] The indiscernible signals from overlap or the complex multiplicity are reported without designating multiplicity.

[b] The data were measured at 600 MHz.

Table S5. NMR assignments for 4 in $CDCl_3$ (^1H at 600 MHz and ^{13}C at 150 MHz).

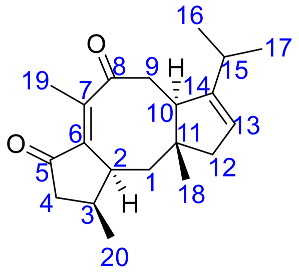

No.	δ_C, type	δ_H (J in Hz)[a]	^1H–^1H COSY	HMBC	NOESY
1	38.2, CH_2	a: 1.68, dd (15.0, 1.8)	1b, 2	2, 3, 6, 10, 11, 12, 18	20
		b: 1.49, dd (15.0, 11.4)	1a, 2	2, 6, 10, 11, 12, 18	18, 20
2	44.7, CH	2.87	1a, 1b, 3, 19	3, 6, 7, 20	4a, 10
3	33.2, CH	2.26	2, 4a, 4b, 20	1, 2, 4, 5, 20	
4	47.0, CH_2	a: 2.49, dd (18.0, 7.8)	3, 4b	3, 5, 20	2
		b: 2.10, dd (18.0, 3.0)	3, 4a	2, 3, 5, 6, 20	20
5	208.7, C				
6	135.0, C				
7	145.1, C				
8	210.4, C				
9	42.2, CH_2	a: 2.75, dd (13.0, 3.8)	9b, 10	8, 10, 11, 14	15, 16
		b: 2.51, t (13.4)	9a, 10	7, 8, 10, 11, 14	18
10	45.9, CH	3.06, br d (13.4)	9a, 9b, 13	8, 9, 11, 13, 14	2, 12a, 16
11	45.9, C				
12	44.7, CH_2	a: 2.26	12b, 13	13, 14, 18	10
		b: 1.88, br d (15.0)	12a, 13		18
13	119.7, CH	5.32, br s	10, 12a, 12b	10, 11, 14, 15	16, 17
14	149.9, C				
15	27.5, CH	2.12	16, 17	13, 14, 16, 17	9a
16	22.0, CH_3	0.99, d (6.6)	15	14, 15, 17	9a, 10, 13
17	21.1, CH_3	1.06, d (6.6)	15	14, 15, 16	13
18	23.0, CH_3	0.97, s		1, 10, 11	1b, 9b, 12b
19	16.5, CH_3	2.27, d (2.4)	2	6, 7, 8	
20	15.3, CH_3	0.85, d (7.2)	3	2, 3, 4	1a, 1b, 4b

[a] The indiscernible signals due to overlap or the complex multiplicity are reported without designating multiplicity.

Supplementary Figures

A)

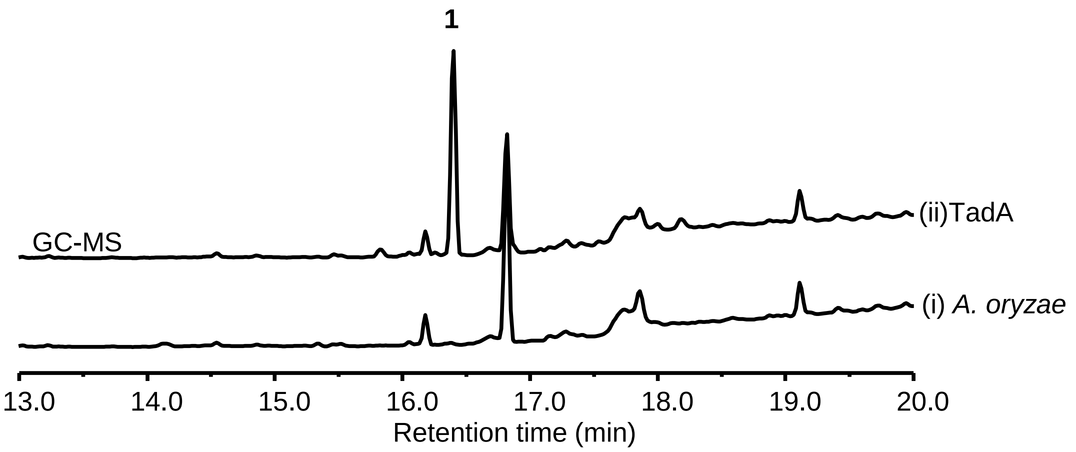

B)

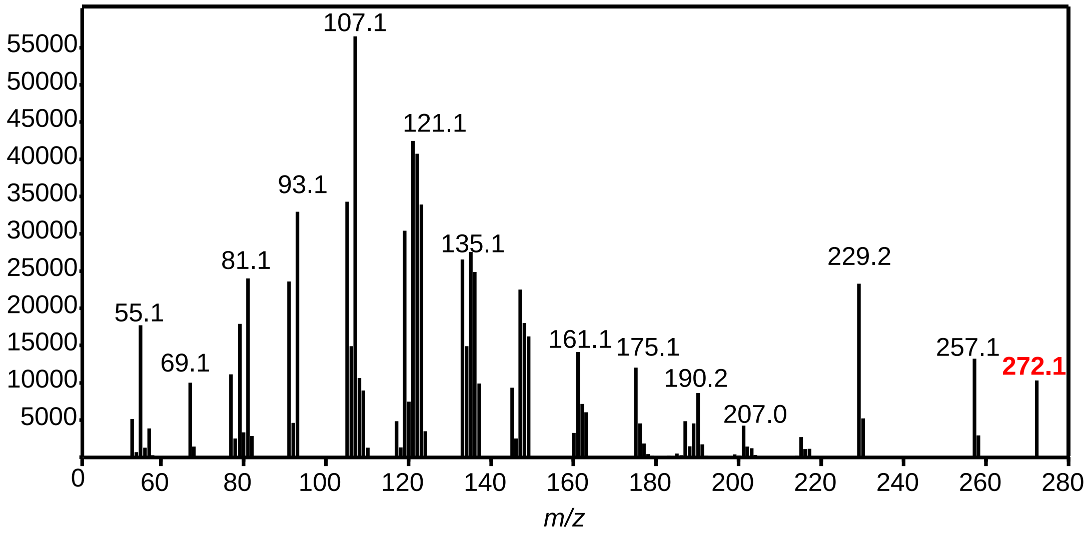

Figure S1. Functional analysis of TadA.

A) GC-MS analysis of metabolites from the *A. oryzae* transformant harboring *tadA*. B) EI mass spectrum of **1**.

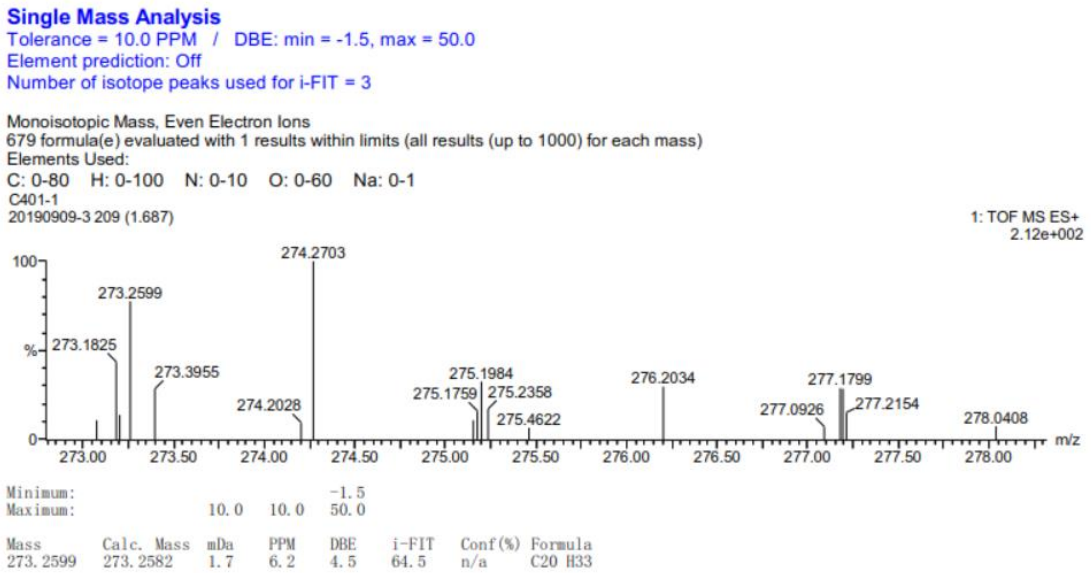

Figure S2. HRESIMS spectrum of 1.

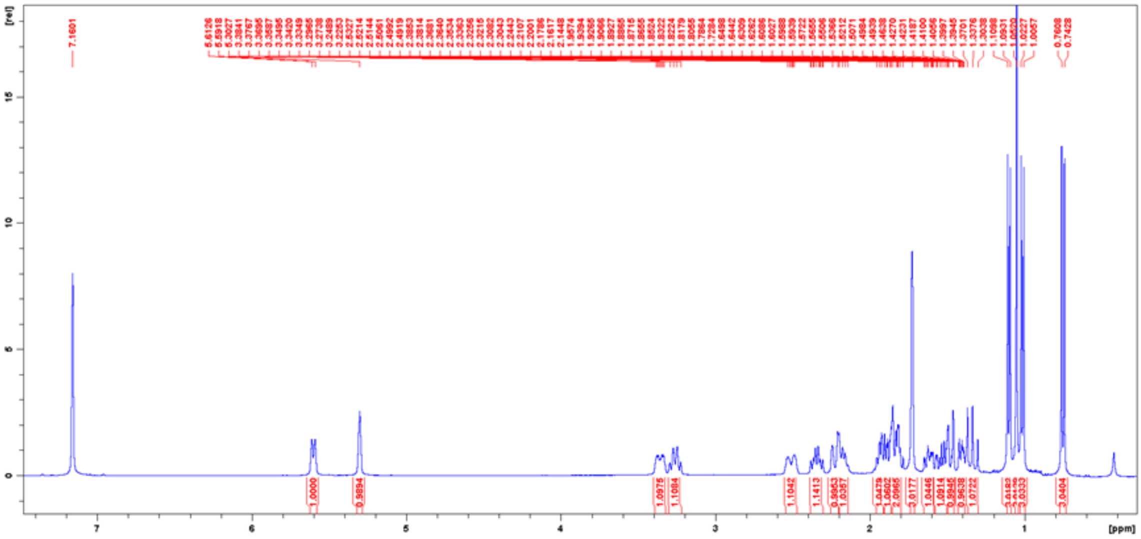

Figure S3. ^{1}H NMR spectrum of 1 in C_6D_6 at 400 MHz.

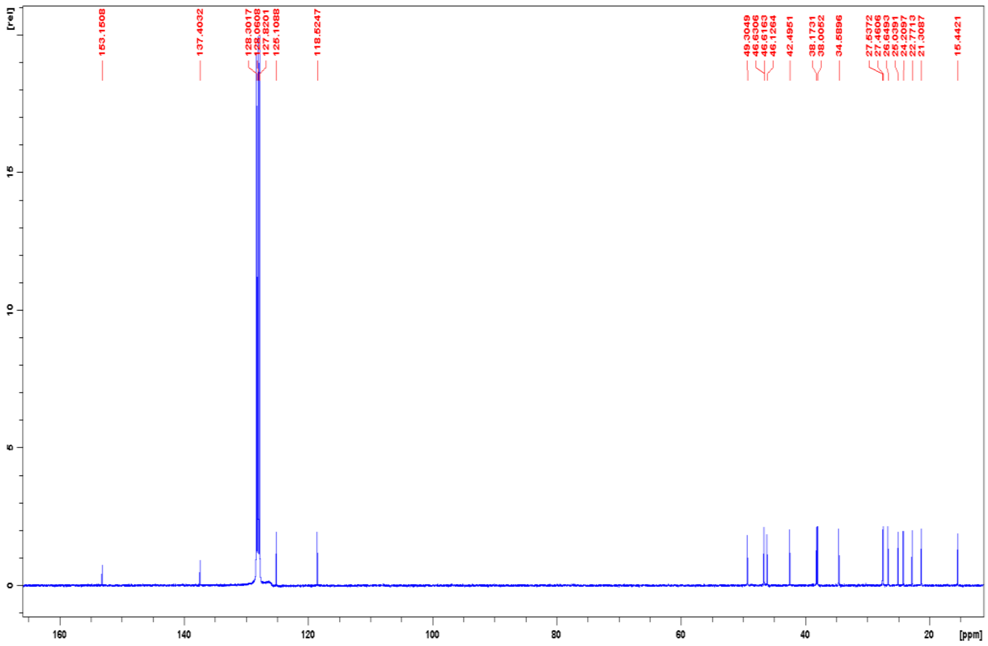

Figure S4. **^{13}C NMR spectrum of 1 in C_6D_6 at 100 MHz.**

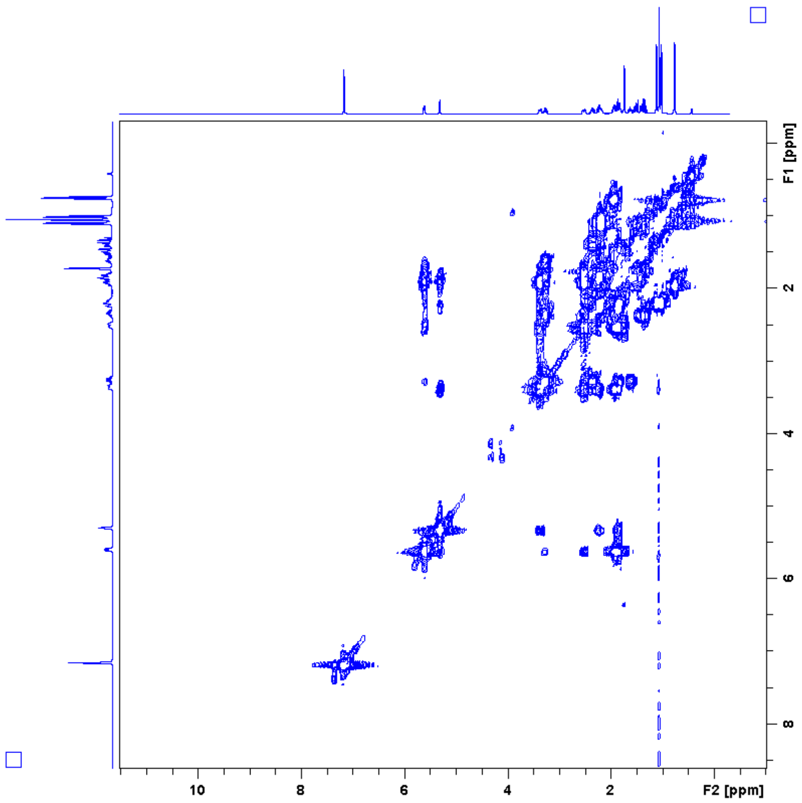

Figure S5. **^{1}H-^{1}H COSY spectrum of 1 in C_6D_6 at 400 MHz.**

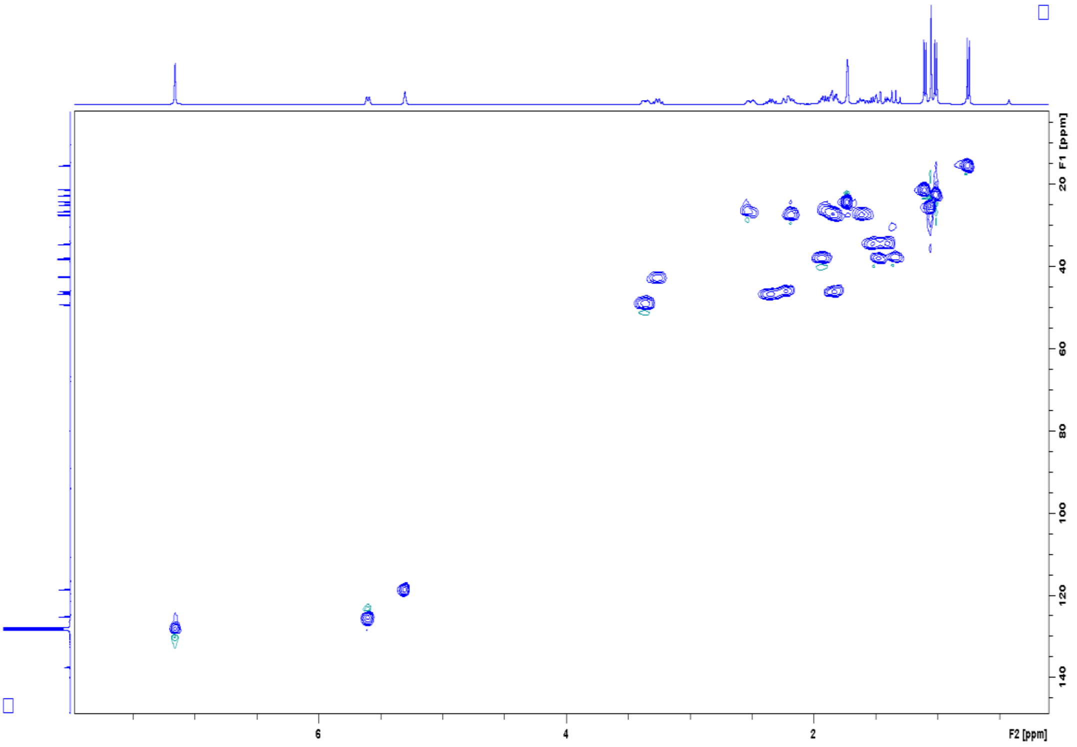

Figure S6. HSQC spectrum of 1 in C_6D_6 at 400 MHz.

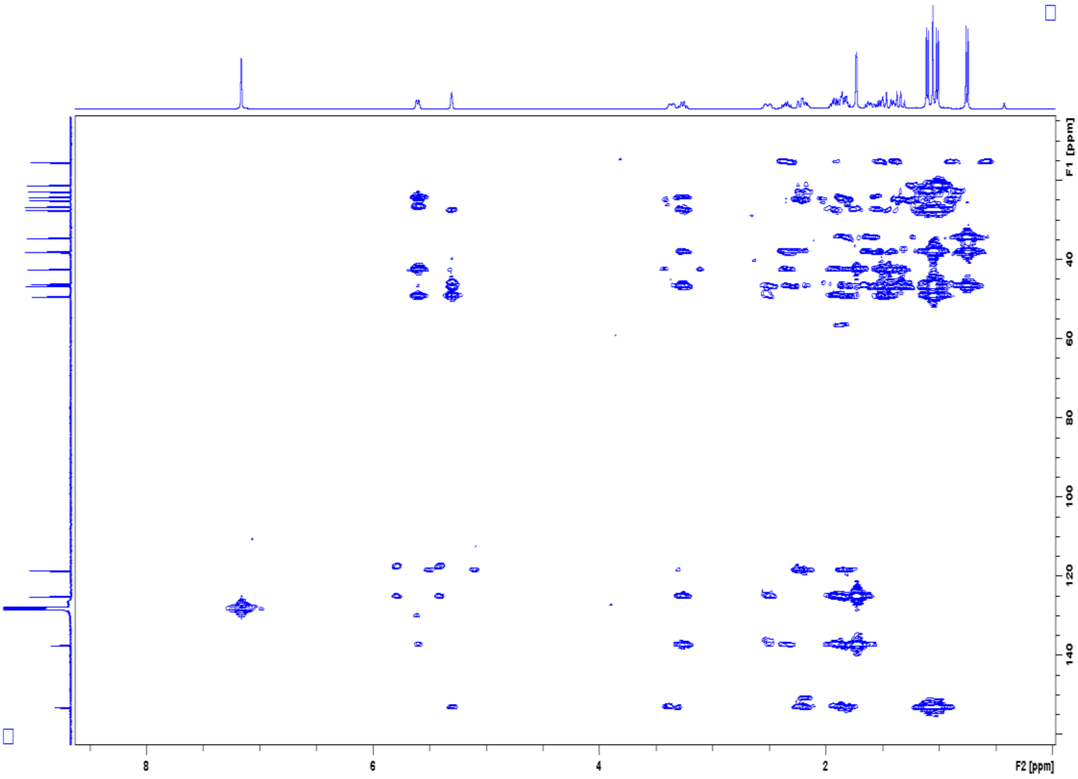

Figure S7. HMBC spectrum of 1 in C_6D_6 at 400 MHz.

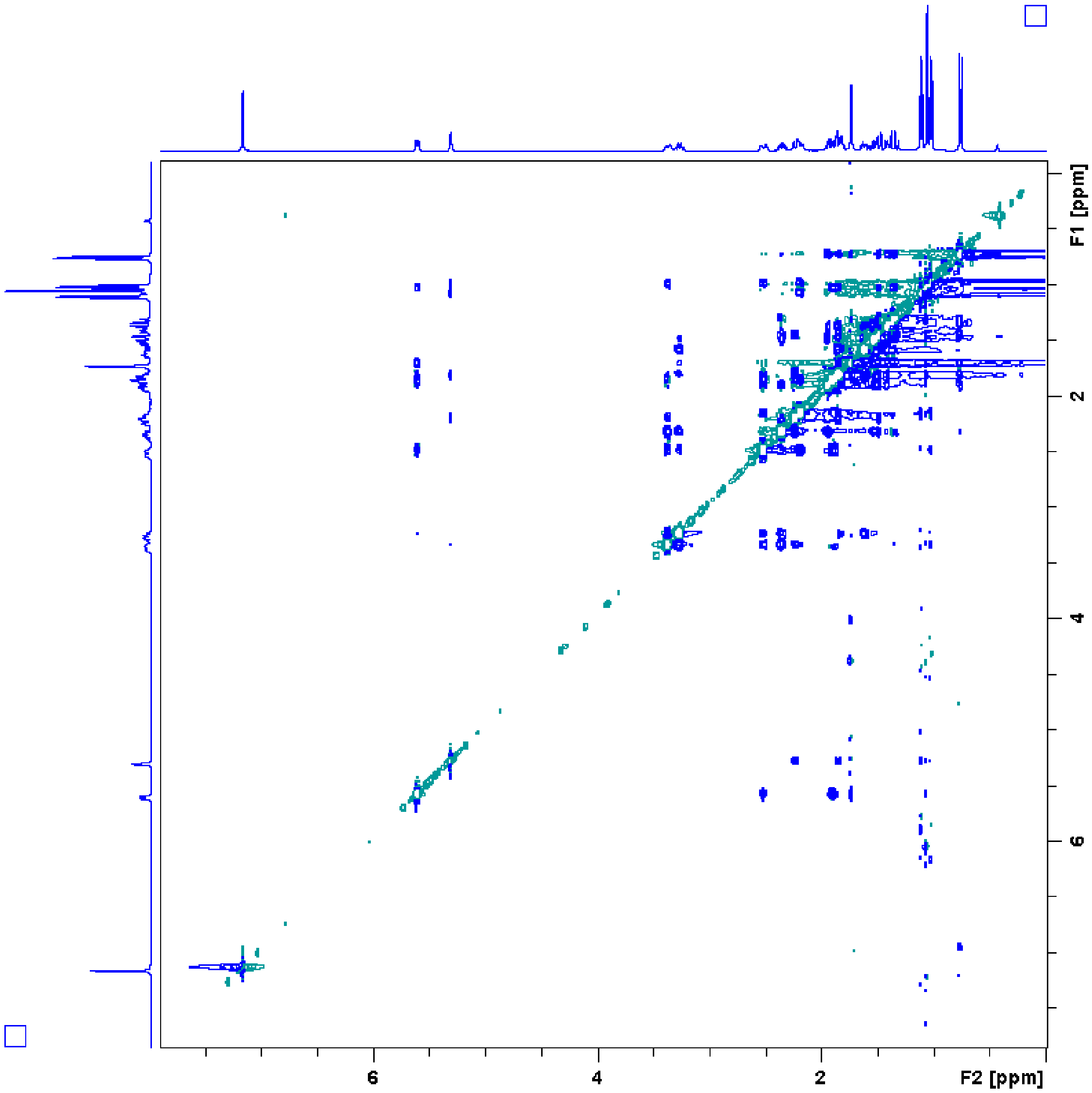

Figure S8. NOESY spectrum of 1 in C_6D_6 at 600 MHz.

1A

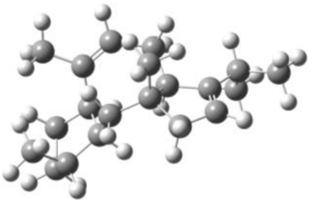

Conformer 1 (79.30%)

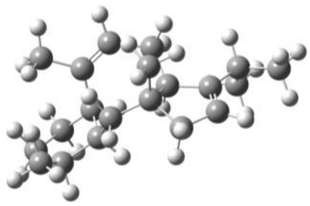

Conformer 2 (7.82%)

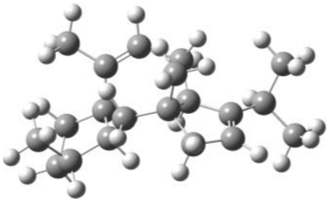

Conformer 3 (6.49%)

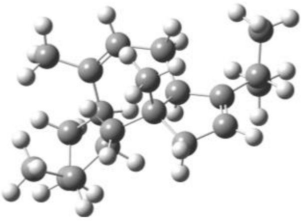

Conformer 4 (6.39%)

1B

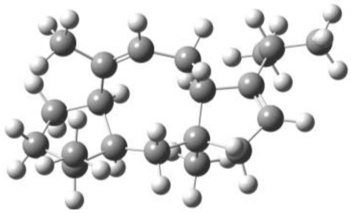

Conformer 1 (87.84%)

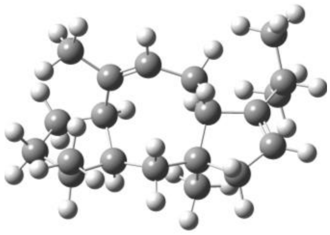

Conformer 2 (12.16%)

Figure S9. Most stable conformers of (2*S, 3*S**, 6*R**, 10*R**, 11*S**)–1A and (2*S**, 3*S**, 6*R**, 10*R**, 11*R**)–1B.**

The relative population is in parenthesis.

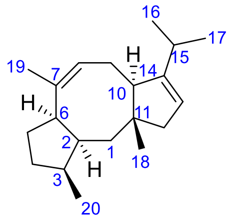

(2*S**, 3*S**, 6*R**, 10*R**, 11*S**)–**1A**

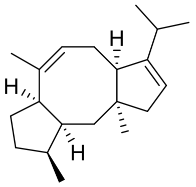

(2*S**, 3*S**, 6*R**, 10*R**, 11*R**)–**1B**

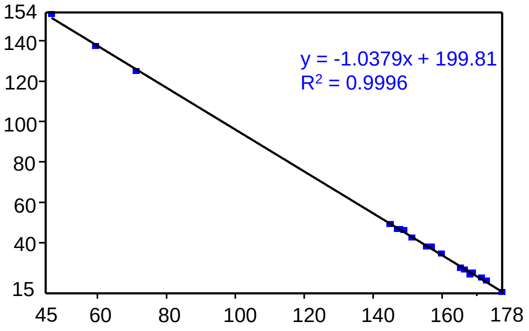

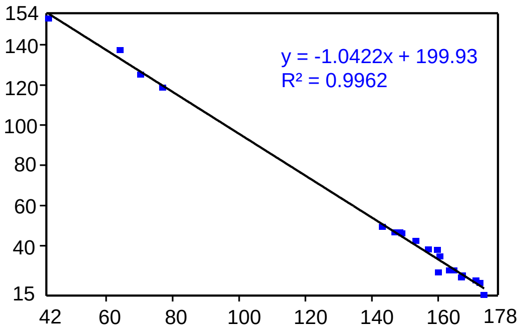

Configuration	1A	1B
Mean absolute error	0.6869	2.1094
DP4+	100%	0%

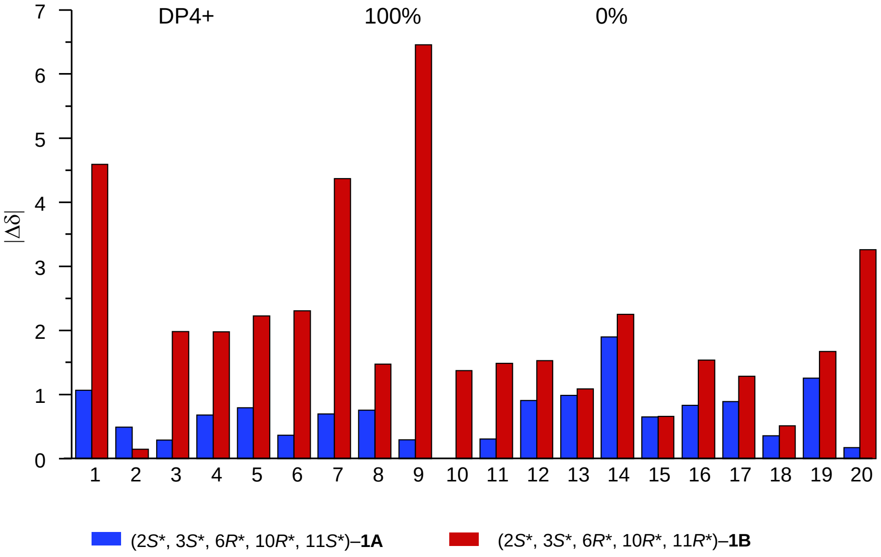

Figure S10. Determination of the relative configuration of C-11 in 1 on the basis of R^2, mean absolute error, and DP4+ probability.

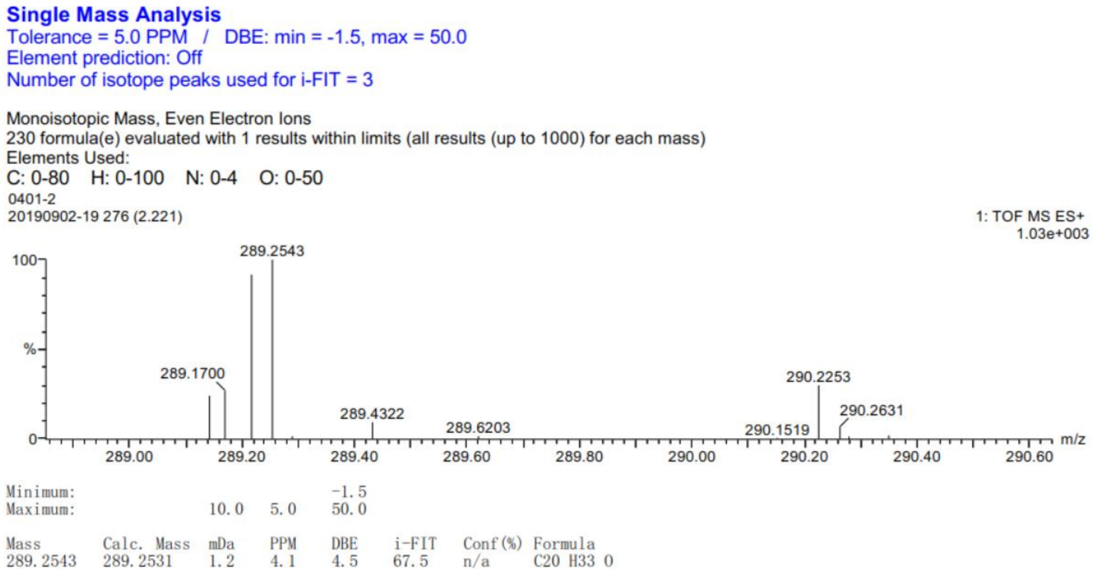

Figure S11. HRESIMS spectrum of 2.

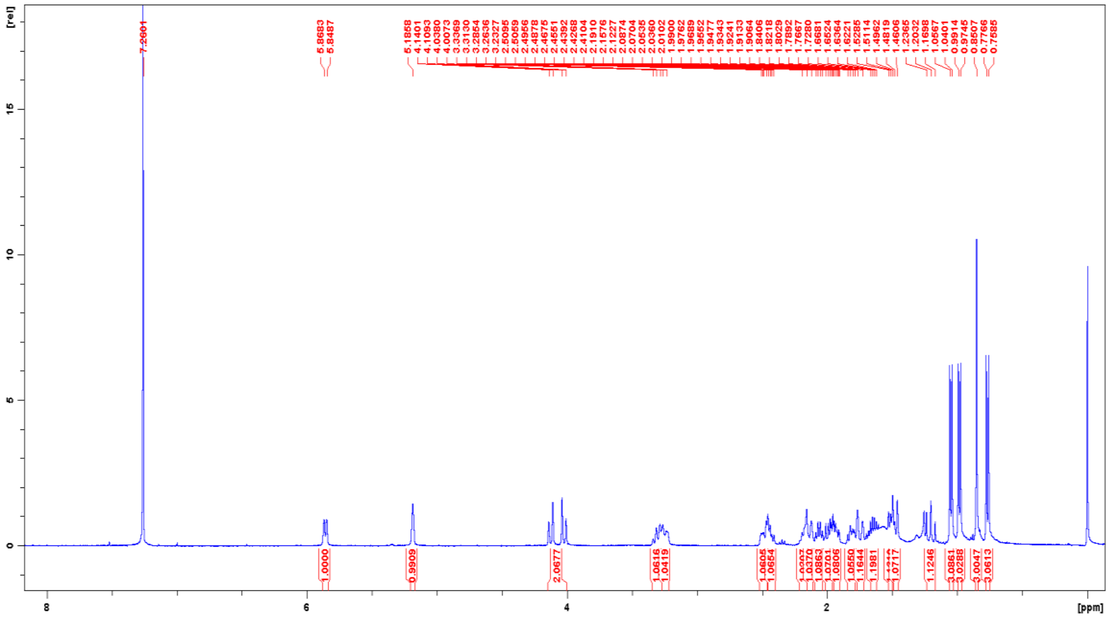

Figure S12. ^{1}H NMR spectrum of 2 in $CDCl_3$ at 400 MHz.

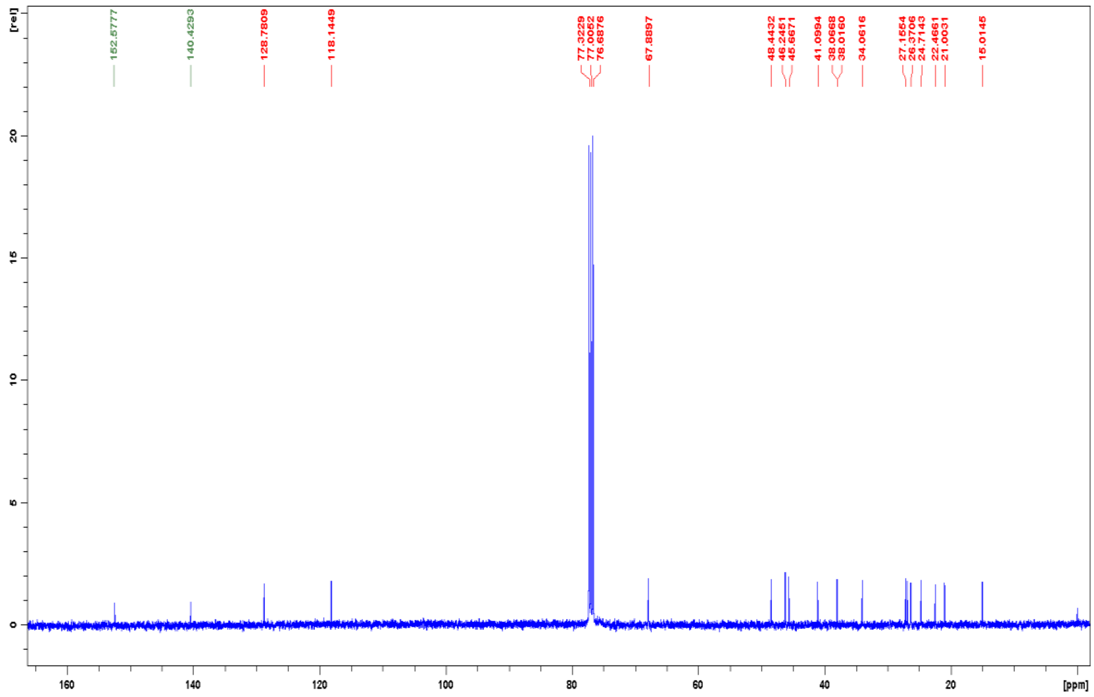

Figure S13. ^{13}C **NMR spectrum of 2 in** $CDCl_3$ **at 100 MHz.**

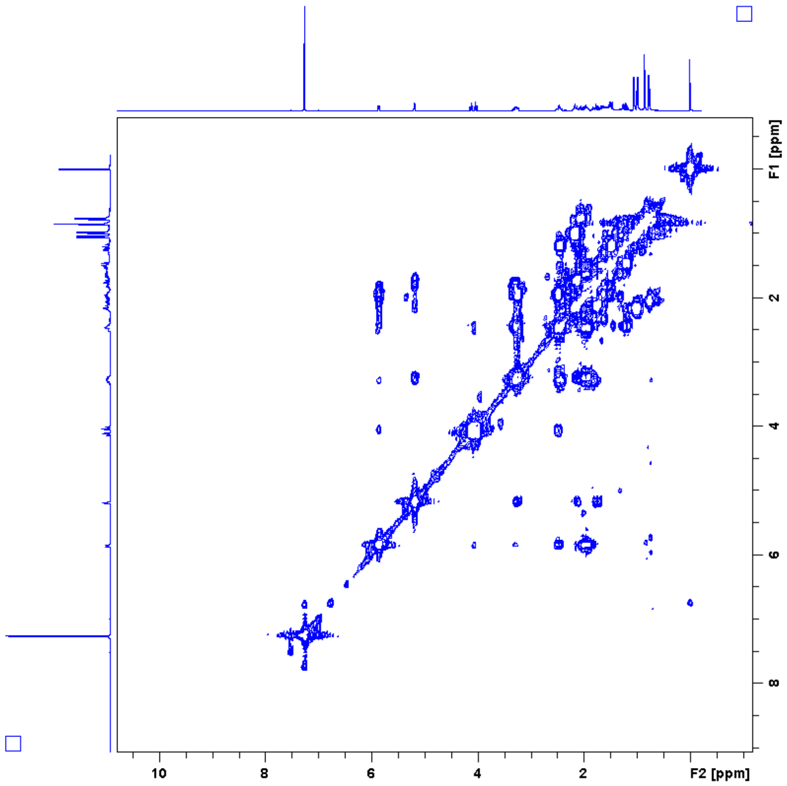

Figure S14. ^{1}H-^{1}H **COSY spectrum of 2 in** $CDCl_3$ **at 400 MHz.**

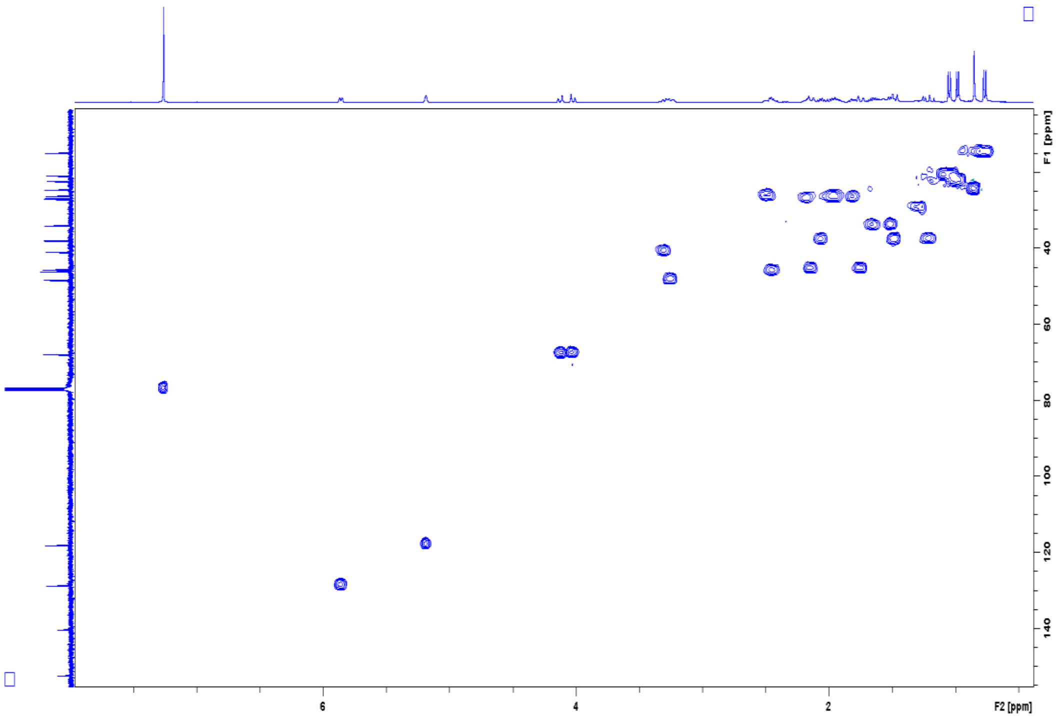

Figure S15. HSQC spectrum of 2 in $CDCl_3$ at 400 MHz.

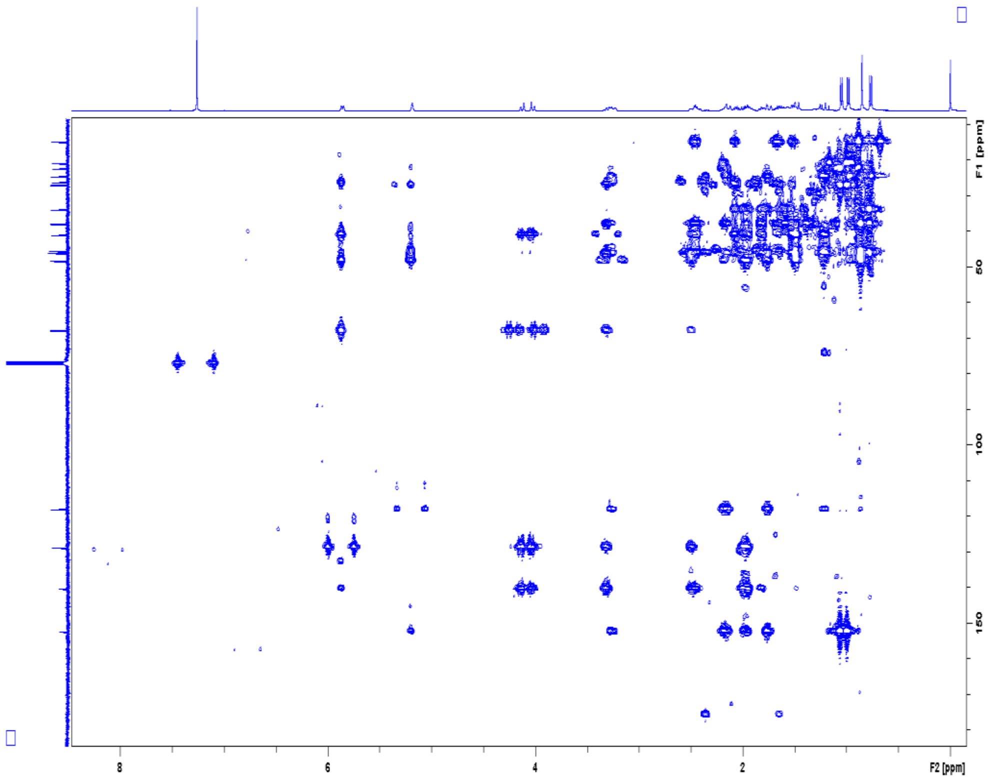

Figure S16. HMBC spectrum of 2 in $CDCl_3$ at 600 MHz.

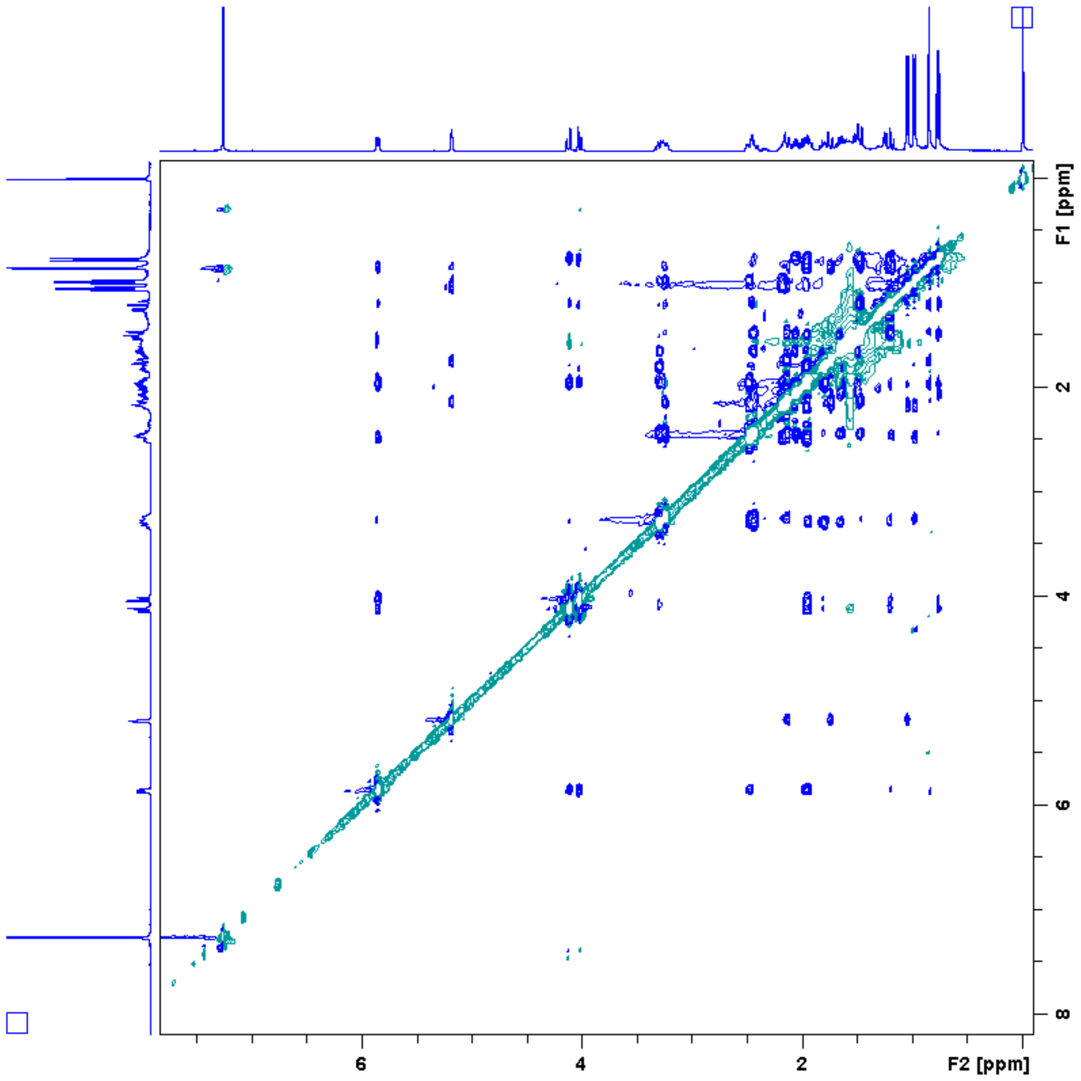

Figure S17. NOESY spectrum of 2 in $CDCl_3$ at 600 MHz.

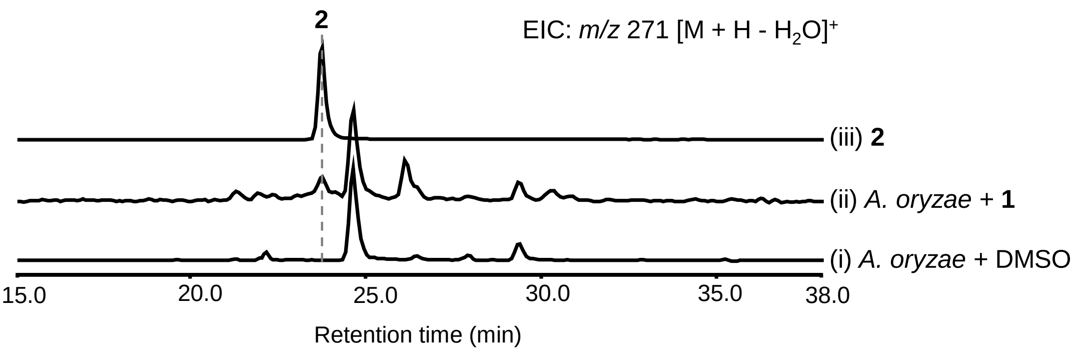

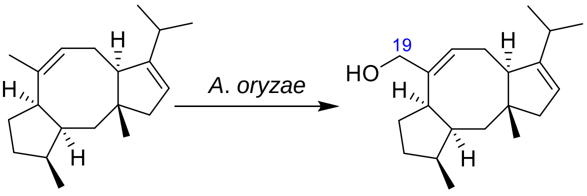

1 **2**

Figure S18. Extracted ion chromatogram (EIC) of metabolites of *A. oryzae* NSAR1 fed with 1 or DMSO.

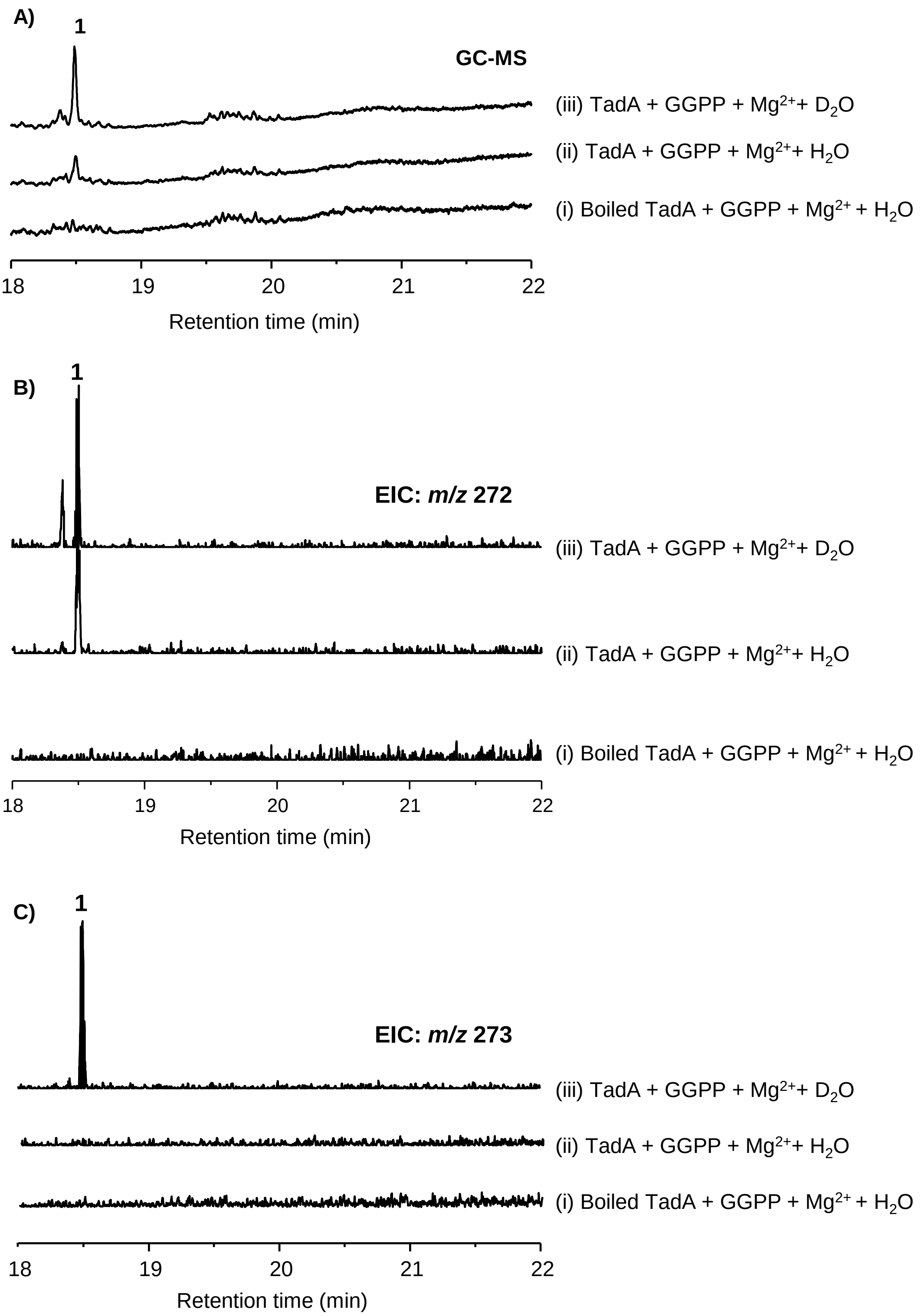

Figure S19. In vitro assay of TadA with addition of D_2O or H_2O.

A) GC-MS analysis. B) EIC at *m/z* 272. C) EIC at *m/z* 273.

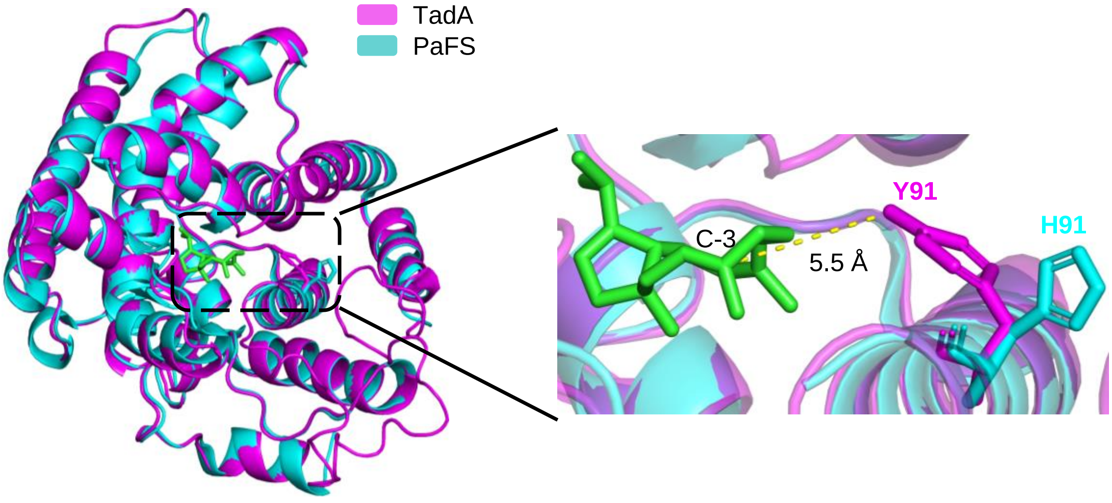

Figure S20. Identification of key amino acid residues for the protonation of the putative neutral intermediate.

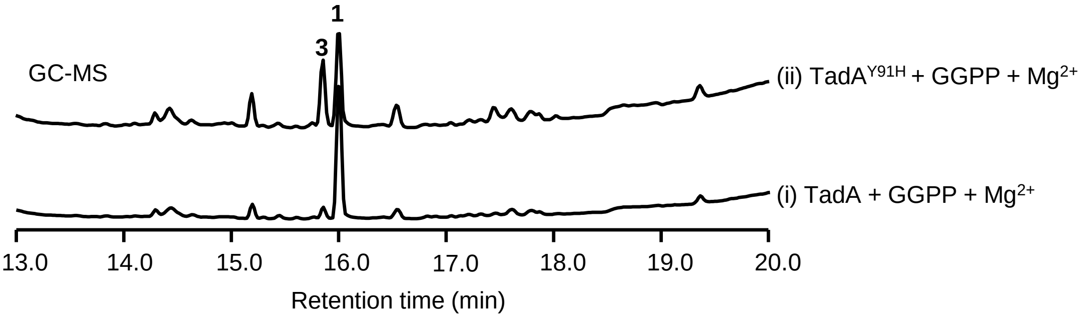

Figure S21. Functional analysis of TadA^{Y91H}.

Soluble fraction of *E.coli* Rosetta(DE3) expressing TadA^{Y91H} or TadA was used for in vitro enzymatic assay.

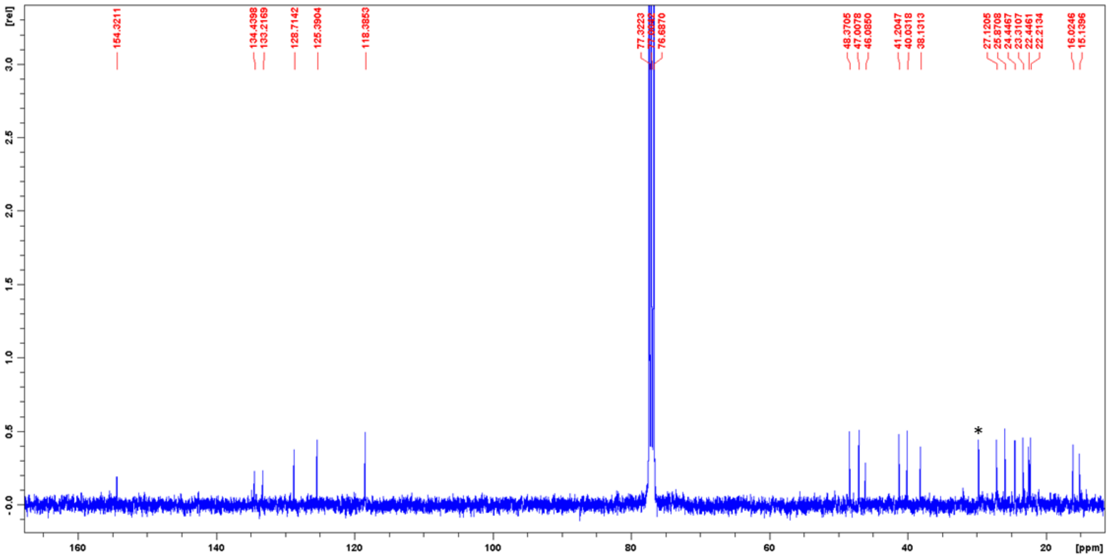

Figure S22. **^{13}C NMR spectrum of 3 in $CDCl_3$ at 100 MHz.**

The signal marked with an asterisk is from grease.

3A

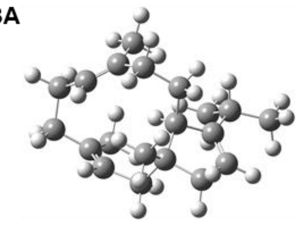

Conformer 1 (88.75%)

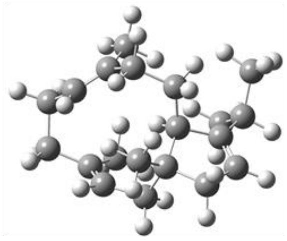

Conformer 2 (3.91%)

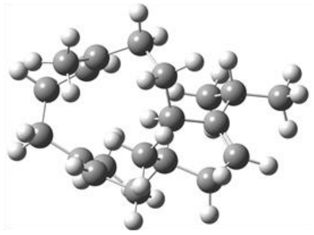

Conformer 3 (1.76%)

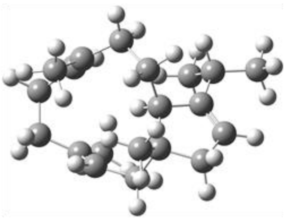

Conformer 4 (1.76%)

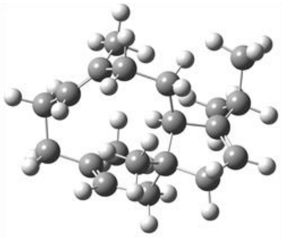

Conformer 5 (3.83%)

3B

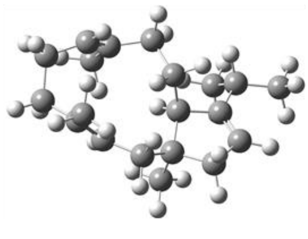

Conformer 1 (31.55%)

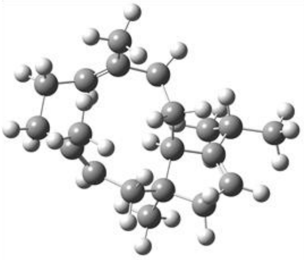

Conformer 2 (14.38%)

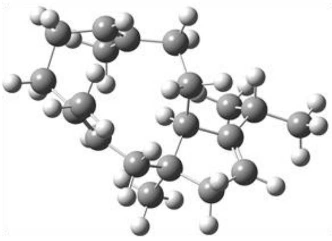

Conformer 3 (31.55%)

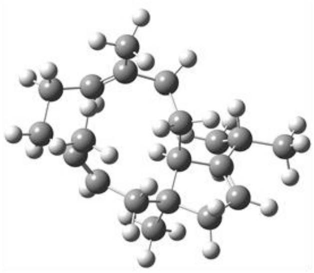

Conformer 4 (14.38%)

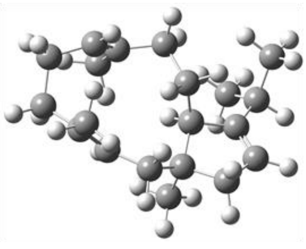

Conformer 5 (4.31%)

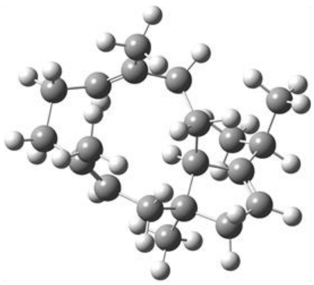

Conformer 6 (2.67%)

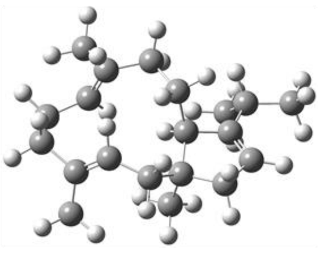

Conformer 7 (1.17%)

Figure S23. Most stable conformers of (10*R,11*S**)-3A and (10*R**,11*R**)-3B.**

The relative population is in parenthesis.

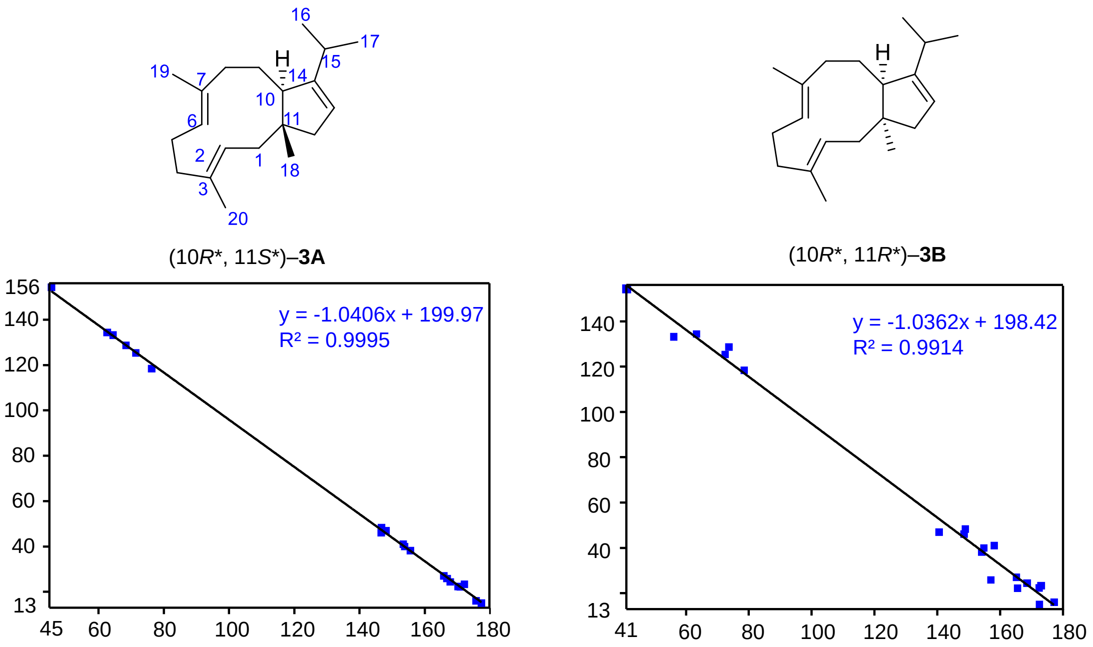

Configuration	3A	3B
Mean absolute error	0.7871	3.4170
DP4+	100%	0%

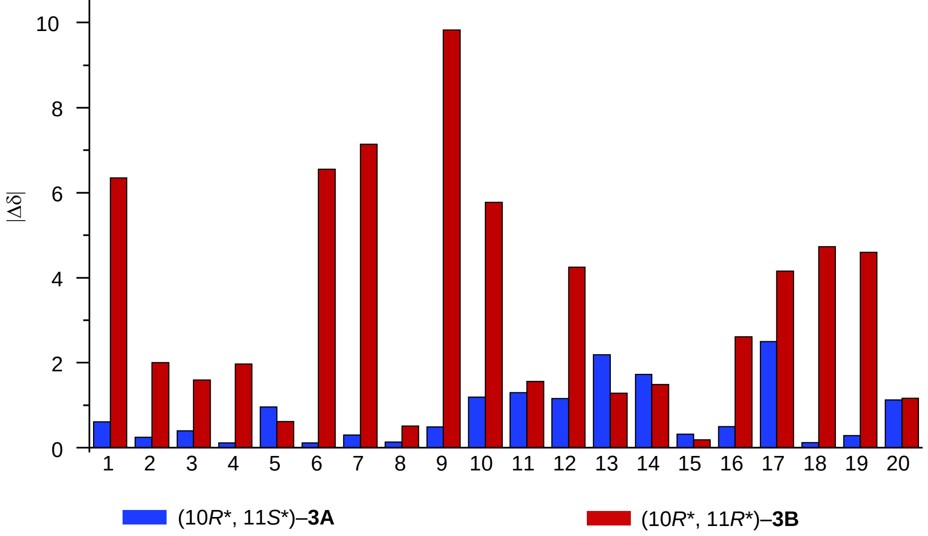

Figure S24. Determination of the relative configuration of 3 on the basis of R^2, mean absolute error, and DP4+ probability.

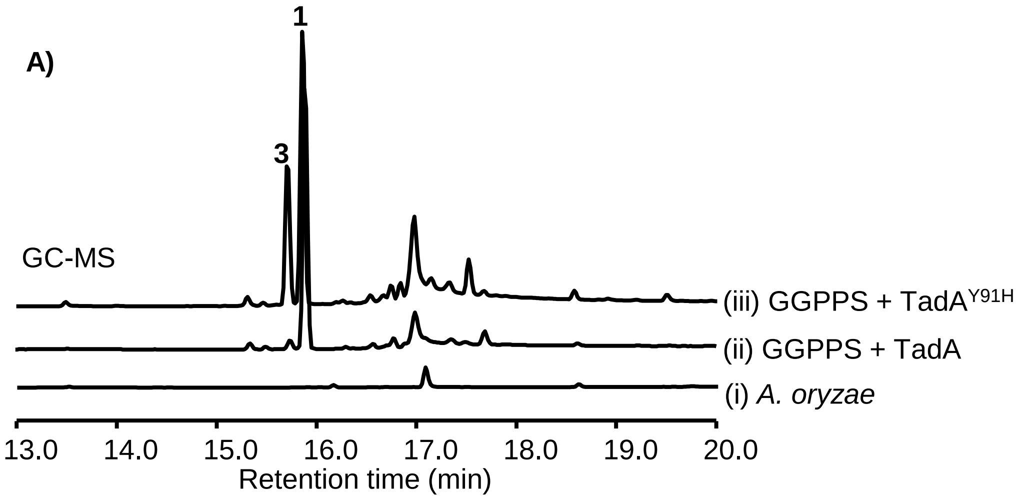

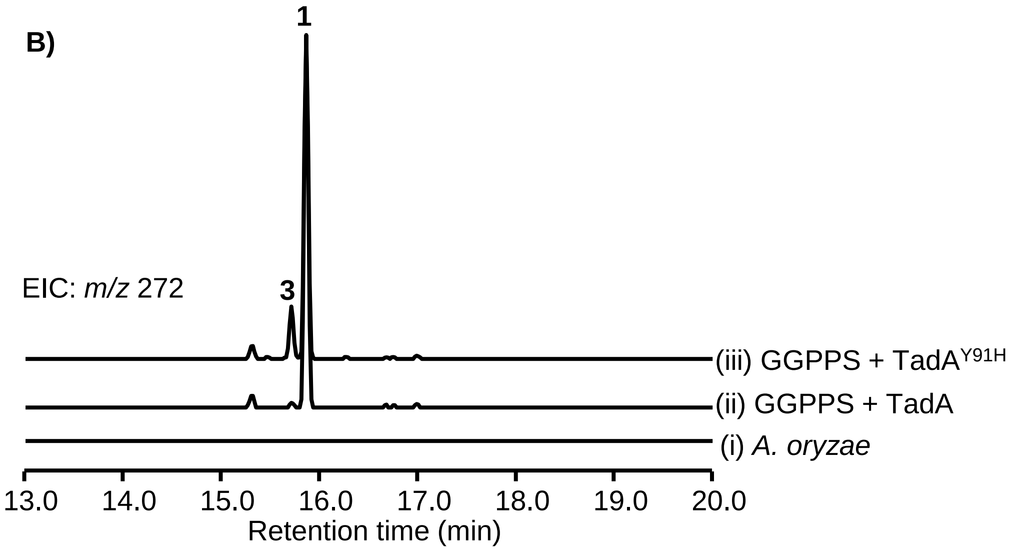

Figure S25. GC-MS analysis of metabolites from *A. oryzae* NSAR1 transformants expressing TadA or TadA^{Y91H}.

(A) GC-MS analysis. (B) EIC at *m/z* 272.

Single Mass Analysis
Tolerance = 5.0 PPM / DBE: min = -1.5, max = 50.0
Element prediction: Off
Number of isotope peaks used for i-FIT = 3

Monoisotopic Mass, Even Electron Ions
245 formula(e) evaluated with 1 results within limits (all results (up to 1000) for each mass)
Elements Used:
C: 0-80 H: 0-100 N: 0-4 O: 0-50
0401-5
20190902-20 284 (2.292)

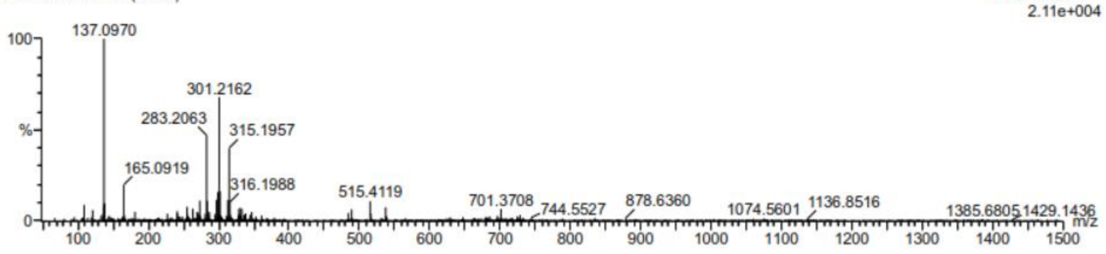

Minimum:				-1.5			
Maximum:		10.0	5.0	50.0			
Mass	Calc. Mass	mDa	PPM	DBE	i-FIT	Conf(%)	Formula
301.2162	301.2168	-0.6	-2.0	6.5	217.9	n/a	C20 H29 O2

Figure S26. HRESIMS spectrum of 4.

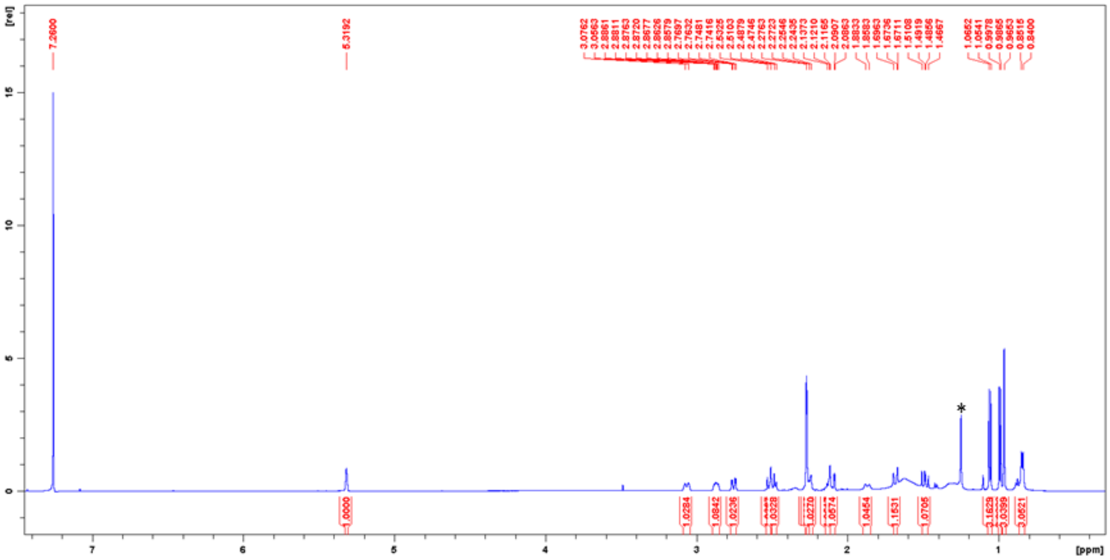

Figure S27. ^1H NMR spectrum of 4 in $CDCl_3$ at 600 MHz.

The signal marked with an asterisk is from grease.

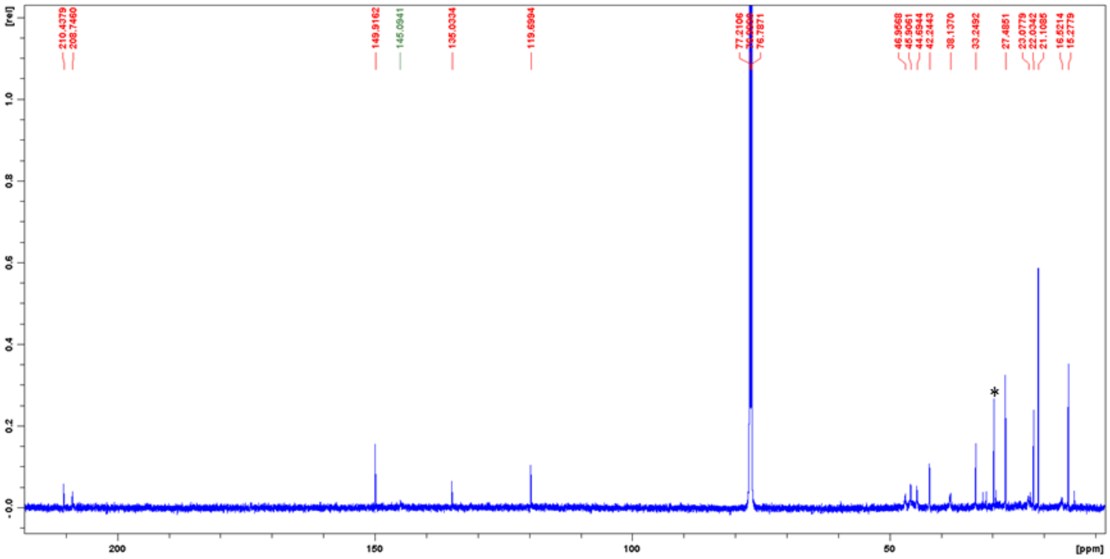

Figure S28. [13]**C NMR spectrum of 4 in $CDCl_3$ at 150 MHz.**

The signal marked with an asterisk is from grease.

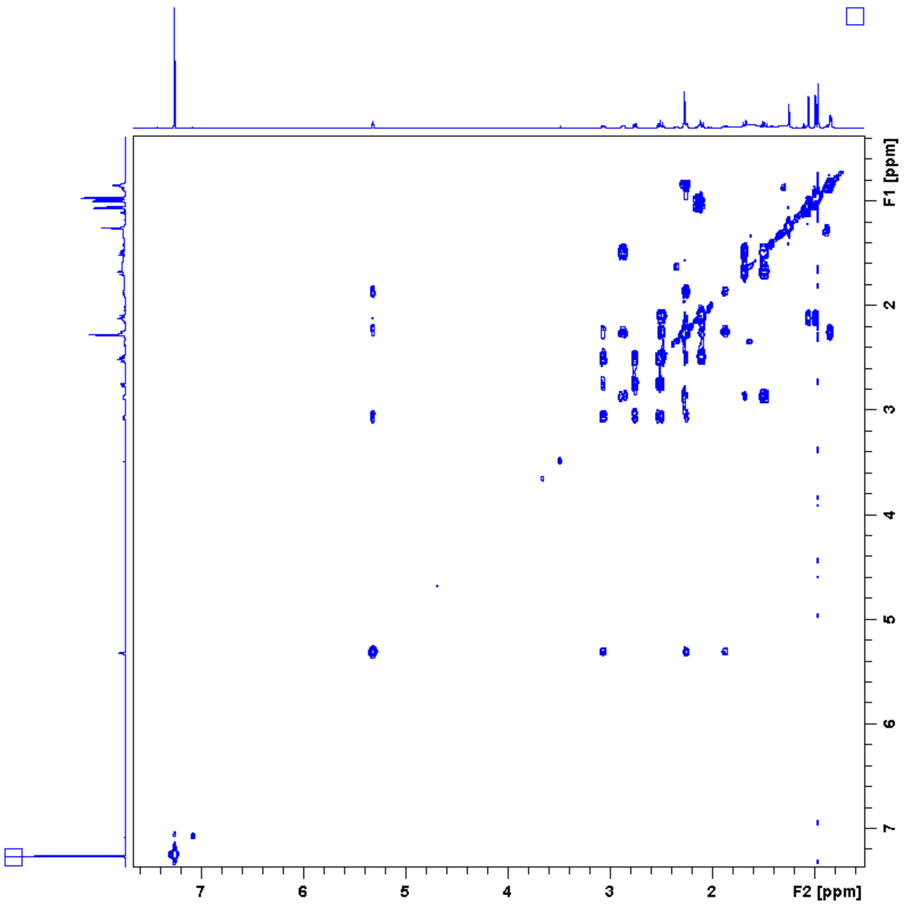

Figure S29. [1]**H-**[1]**H COSY spectrum of 4 in $CDCl_3$ at 600 MHz.**

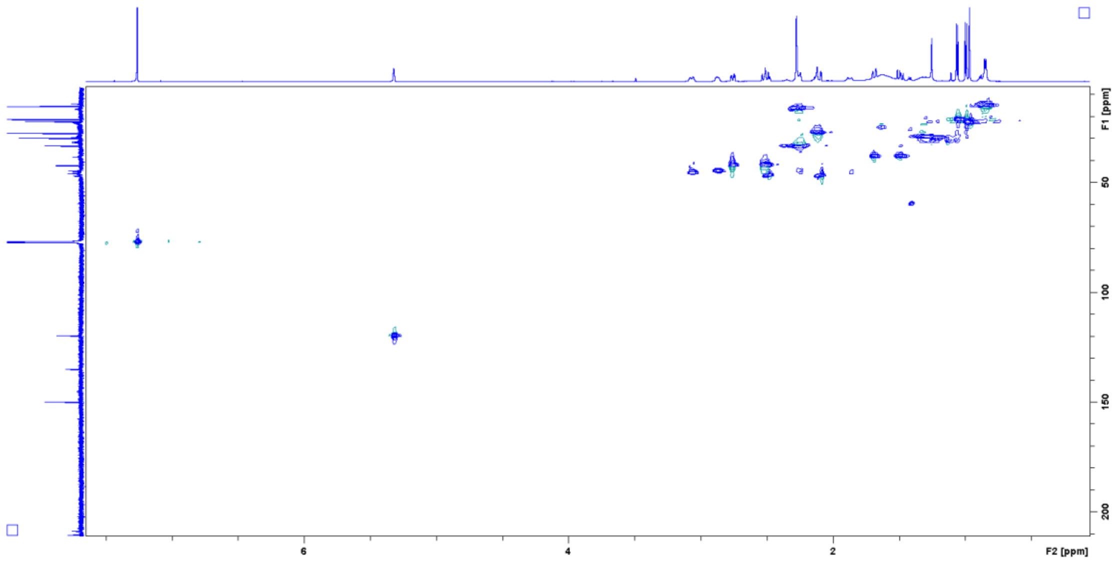

Figure S30. HSQC spectrum of 4 in $CDCl_3$ at 600 MHz.

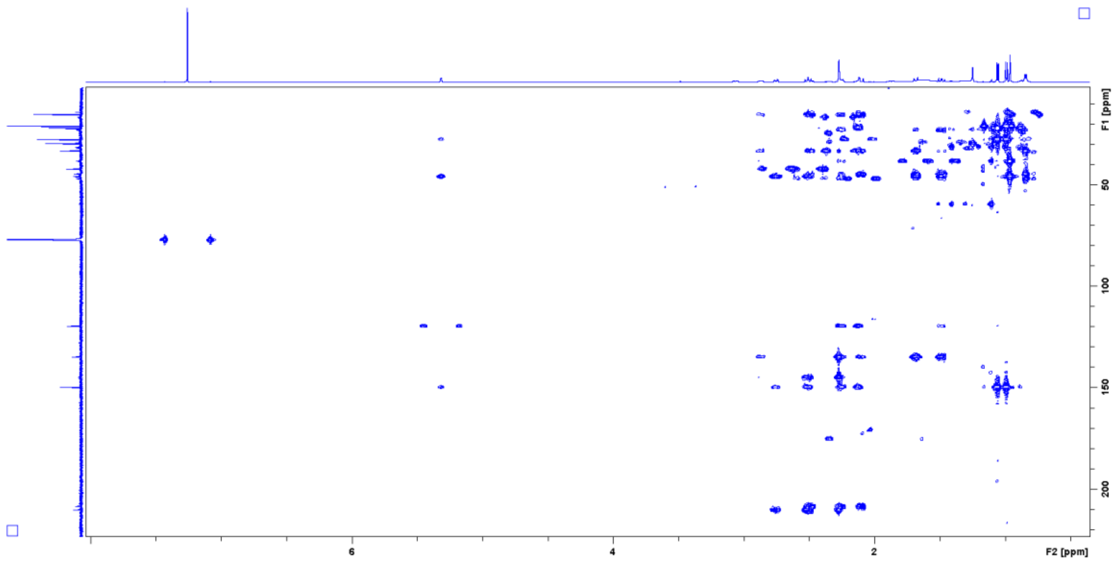

Figure S31. HMBC spectrum of 4 in $CDCl_3$ at 600 MHz.

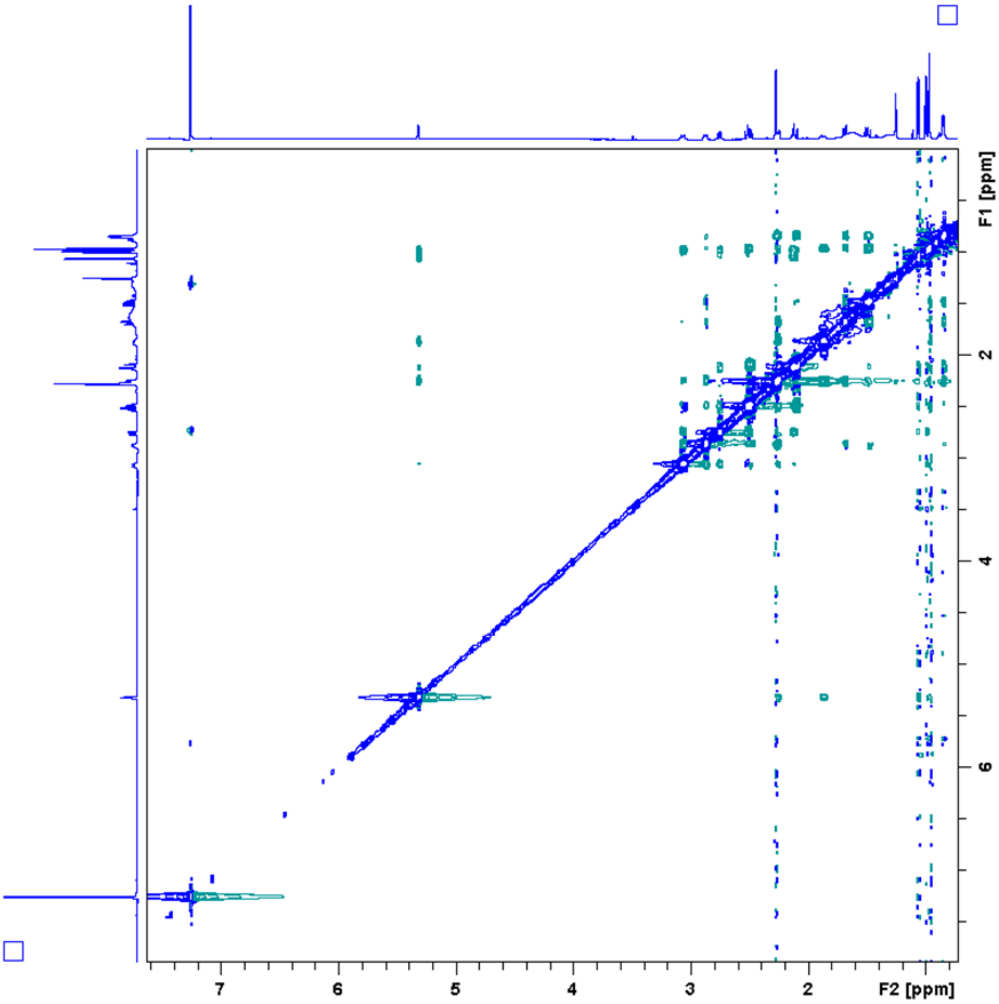

Figure S32. NOESY spectrum of 4 in $CDCl_3$ at 600 MHz.

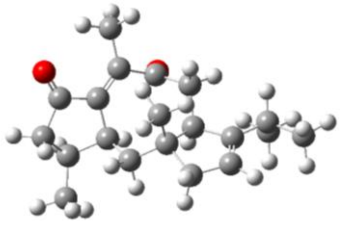

Conformer 1 (37.36%)

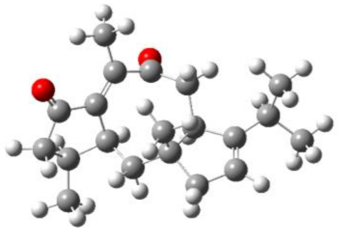

Conformer 2 (4.32%)

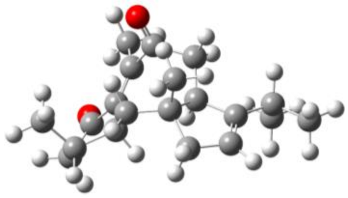

Conformer 3 (45.85%)

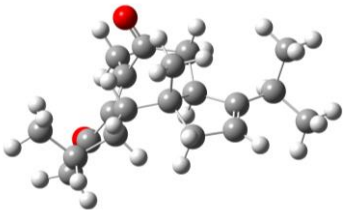

Conformer 4 (6.95%)

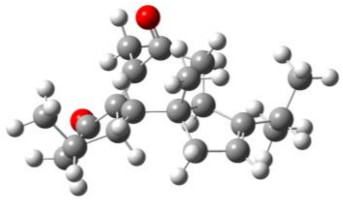

Conformer 5 (5.53%)

Figure S33. Most stable conformers of (2*S*,3*S*,10*R*,11*R*)-4.

The relative population is in parenthesis.

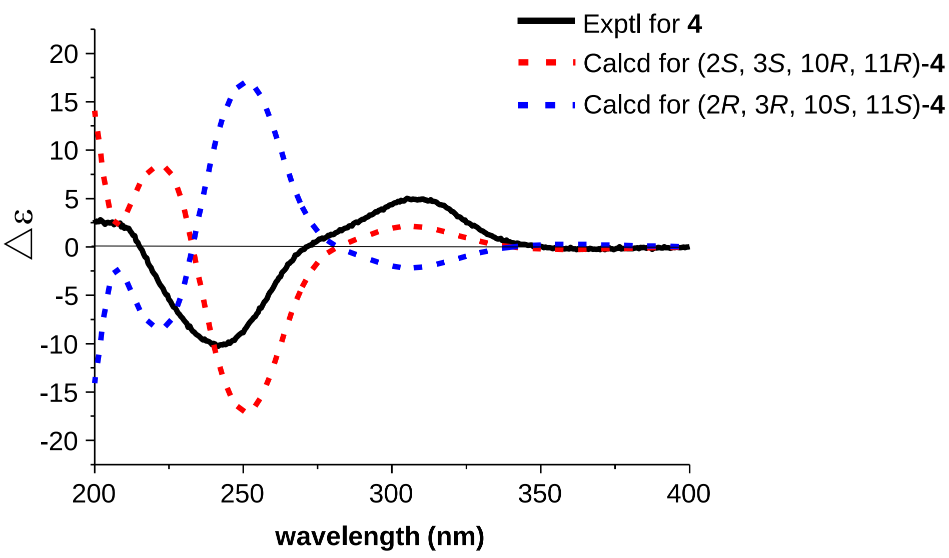

Figure S34. Comparison of experimental ECD spectrum of 4 and calculated ECD spectra of (2*S*,3*S*,10*R*,11*R*)-4/(2*R*,3*R*,10*S*,11*S*)-4.

UV correction = +16.1 nm; band width σ = 0.3 eV.

Supplementary References

[1]. Jin, F. J.; Maruyama, J.; Juvvadi, P. R.; Arioka, M.; Kitamoto, K. *FEMS Microbiol. Lett.* **2004**, *239*, 79-85. doi:10.1016/j.femsle.2004.08.025

[2]. Wang, G. Q.; Chen, G. D.; Qin, S. Y.; Hu, D.; Awakawa, T.; Li, S. Y.; Lv, J. M.; Wang, C. X.; Yao, X. S.; Abe, I.; Gao, H. *Nat. Commun.* **2018**, *9*. doi:10.1038/s41467-018-04298-2

[3]. Trott, O.; Olson, A. J. *J Comput Chem* **2010**, *31*, 455-461. doi:10.1002/jcc.21334

[4]. *Gaussian 09*, Revision B.01; Gaussian, Inc.: Wallingford, CT, 2009

[5]. Grimblat, N.; Zanardi, M. M.; Sarotti, A. M. *J. Org. Chem.* **2015**, *80*, 12526-12534. doi:10.1021/acs.joc.5b02396

[6]. Bruhn, T.; Schaumloffel, A.; Hemberger, Y.; Bringmann, G. *Chirality* **2013**, *25*, 243-249. doi:10.1002/chir.22138

[7]. Ioannou, E.; Quesada, A.; Rahman, M. M.; Gibbons, S.; Vagias, C.; Roussis, V. *Eur. J. Org. Chem.* **2012**, *2012*, 5177-5186. doi:10.1002/ejoc.201200533

[8]. Fujii, T.; Yamaoka, H.; Gomi, K.; Kitamoto, A.; Kumagai, C. *Biosci. Biotechnol. Biochem.* **1995**, *59*, 1869-1874. doi:10.1271/bbb.59.1869

[9]. Jin, F. J.; Maruyama, J.; Juvvadi, P. R.; Arioka, M.; Kitamoto, K. *Biosci. Biotechnol. Biochem.* **2004**, *68*, 656-662. doi:10.1271/bbb.68.656

[10]. Lin, F. L.; Lauterbach, L.; Zou, J.; Wang, Y. H.; Lv, J. M.; Chen, G. D.; Hu, D.; Gao, H.; Yao, X. S.; Dickschat, J. S. *ACS Catal.* **2020**, *10*, 4306-4312. doi:10.1021/acscatal.0c00377
